# Homologues of potato chromosome 5 show variable collinearity in the euchromatin, but dramatic absence of sequence similarity in the pericentromeric heterochromatin

**DOI:** 10.1186/s12864-015-1578-1

**Published:** 2015-05-10

**Authors:** Jan M de Boer, Erwin Datema, Xiaomin Tang, Theo J A Borm, Erin H Bakker, Herman J van Eck, Roeland C H J van Ham, Hans de Jong, Richard G F Visser, Christian W B Bachem

**Affiliations:** Wageningen UR Plant Breeding, Wageningen University and Research Centre, Droevendaalsesteeg 1, 6708PB Wageningen, The Netherlands; Wageningen University and Research Centre, Applied Bioinformatics, Plant Research International, Droevendaalsesteeg 1, 6708PB Wageningen, The Netherlands; Laboratory of Genetics, Wageningen University, Droevendaalsesteeg 1, 6708PB Wageningen, The Netherlands; Laboratory of Nematology, Wageningen University, Droevendaalsesteeg 1, 6708PB Wageningen, The Netherlands; Current address: Averis Seeds B.V., Valtherblokken Zuid 40, 7876 TC Valthermond, The Netherlands; Current address: KeyGene N.V., P.O. Box 216, 6700 Wageningen, The Netherlands; Current address: Department of Biology, Colorado State University, Fort Collins, USA

## Abstract

**Background:**

In flowering plants it has been shown that *de novo* genome assemblies of different species and genera show a significant drop in the proportion of alignable sequence. Within a plant species, however, it is assumed that different haplotypes of the same chromosome align well. In this paper we have compared three *de novo* assemblies of potato chromosome 5 and report on the sequence variation and the proportion of sequence that can be aligned.

**Results:**

For the diploid potato clone RH89-039-16 (RH) we produced two linkage phase controlled and haplotype-specific assemblies of chromosome 5 based on BAC-by-BAC sequencing, which were aligned to each other and compared to the 52 Mb chromosome 5 reference sequence of the doubled monoploid clone DM 1–3 516 R44 (DM). We identified 17.0 Mb of non-redundant sequence scaffolds derived from euchromatic regions of RH and 38.4 Mb from the pericentromeric heterochromatin. For 32.7 Mb of the RH sequences the correct position and order on chromosome 5 was determined, using genetic markers, fluorescence *in situ* hybridisation and alignment to the DM reference genome. This ordered fraction of the RH sequences is situated in the euchromatic arms and in the heterochromatin borders. In the euchromatic regions, the sequence collinearity between the three chromosomal homologs is good, but interruption of collinearity occurs at nine gene clusters. Towards and into the heterochromatin borders, absence of collinearity due to structural variation was more extensive and was caused by hemizygous and poorly aligning regions of up to 450 kb in length. In the most central heterochromatin, a total of 22.7 Mb sequence from both RH haplotypes remained unordered. These RH sequences have very few syntenic regions and represent a non-alignable region between the RH and DM heterochromatin haplotypes of chromosome 5.

**Conclusions:**

Our results show that among homologous potato chromosomes large regions are present with dramatic loss of sequence collinearity. This stresses the need for more *de novo* reference assemblies in order to capture genome diversity in this crop. The discovery of three highly diverged pericentric heterochromatin haplotypes within one species is a novelty in plant genome analysis. The possible origin and cytogenetic implication of this heterochromatin haplotype diversity are discussed.

**Electronic supplementary material:**

The online version of this article (doi:10.1186/s12864-015-1578-1) contains supplementary material, which is available to authorized users.

## Background

Plant genome sequencing has shown an exponential increase in the past decade, with over 50 flowering plant species having been sequenced to date [1]. The majority of these sequencing projects have used inbred lines or genotypes with a naturally occurring low level of polymorphism, which greatly facilitates their whole genome assembly. This list includes a doubled monoploid line that was used to assemble the current potato reference genome [2]. In addition, a limited number of heterozygous diploid genomes have been sequenced as well. For example, the whole genome shotgun (WGS) approach was used for assembling the poplar, grape, apple, and date palm genomes [3-6], whereas high throughput sequencing of bacterial artificial chromosome (BAC) clones was applied for the pear genome [7]. In these heterozygous genome assemblies, the separation of both sequence haplotypes proved to be difficult, or was not attempted. In poplar, much of the heterozygosity-related sequence polymorphism was found to condense into mosaic sequence during the assembly process [8], while in grape small WGS contigs of the alternative haplotype were identified [4]. The quantification of sequence polymorphism in these heterozygous assemblies involved measuring the frequency of single nucleotide polymorphisms (SNPs), short insertions/deletions (indels), and small gaps.

Potato is an open-pollinating species with a highly heterozygous genome. This sequence diversity of the potato genome has been long documented through the characterization of SNPs in its transcriptome, either by bioinformatic mining of existing expressed sequence tag databases [9,10] or by active sequencing of cDNAs and selected genes in cultivar panels [11-13]. Also copy number variation has been shown to contribute to potato sequence diversity [14]. The heterozygosity of the potato genome has been well exploited for the development of genetic maps based on either AFLP or SNP technologies [15-18].

The cultivated potato (*Solanum tuberosum*) is a tetraploid species with tetrasomic chromosome pairing, in which the twelve chromosomes have a haploid genome size of 840 Mb [19]. This means that potato can have up to four different haplotypes. Reconstruction of these different chromosomal haplotypes in a potato genome can be achieved through the phasing of haplotype specific sequence variants. In tetraploid genetic maps, four linkage phases are identified per chromosome (or linkage group), each corresponding to one of the four chromosomal homologs [15]. In diploid potato lines, this phasing simplifies to two homologs per linkage group [16]. The linkage phase specificity of potato genetic markers can be used to assign genomic sequences to their respective homologous chromosomes and thus phase the sequence haplotypes, as was shown for the BAC physical map of the diploid potato clone RH [20].

Potato chromosomes have a well-defined structure that becomes visible during the pachytene stage of meiosis. Each chromosome is composed of two distal euchromatic arms separated by a central pericentromeric region [21,22]. Chromosome 2 lacks the north euchromatic arm, which has been replaced by the nucleolar organizer region [20]. In potato, the cytogenetic distribution of genetically anchored BAC sequences has been explored by fluorescence *in situ* hybridisation (FISH) to pachytene chromosomes, which has resulted in the development a genome-wide set of karyotype markers [22] and a detailed cytogenetic map of chromosome 6 [21].

The Potato Genome Sequencing Consortium (PGSC) has published the potato reference genome [2], which is a WGS assembly from the doubled monoploid clone DM 1–3 516 R44 (*Solanum tuberosum* Group Phureja) which is referred to as DM throughout this text. In addition, the PGSC has also sequenced approximately 10 percent of the heterozygous diploid clone RH89-039-16 (referred to as RH), using BAC clones that were selected from the AFLP fingerprint physical map of this genotype [2,23]. Clone RH differs from DM in that it has a 75% *S. tuberosum* Group Tuberosum background in its pedigree. In a preliminary analysis of potato genome heterozygosity, only regions of close collinear alignment were examined, where the sequence diversity is limited to SNPs and short indels [2]. It was shown that the available RH BAC sequence scaffolds have a 97.5% sequence identity with the DM reference genome. Similarly, an overall 96.5% sequence identity was determined between overlapping RH sequences of opposite haplotypes.

In the present paper, we describe in detail the results from the PGSC BAC sequencing of the two haplotypes of RH chromosome 5 (Chr-5). Potato chromosome 5 was chosen as a target for BAC sequencing because it contains many well-studied trait loci, such as H1, R1, *StCDF* and *StSP6A* [24-27]. Chr-5 is by far the most complete chromosome sequence of genotype RH. The locations of the RH Chr-5 BAC tiling path sequences are presented in relation to the DM reference genome and the RH genetic map. A detailed new Chr-5 cytogenetic map is also presented. Through graphical sequence alignments, the RH Chr-5 sequences were compared to each other and to the DM pseudomolecule reference sequence [18], in order to examine the regional variation in sequence similarity and collinearity. Our results give new insight in the long-distance sequence variation that can be found in the potato genome and are revealing a very high level of plant genome plasticity.

## Results

### BAC sequencing

From Chr-5 of the heterozygous diploid potato clone RH89-039-16 (RH), we have sequenced 597 BAC clones with a total length of 70.2 Mb (Table 1). These partially overlapping BAC sequences were condensed into 55.4 Mb of non-redundant sequence, which is organised into 107 BAC minimal tiling paths (MTPs) for which the statistics are given in Table 2, Additional file 1: Table S1 and Additional file 2: Table S2. The tiling path sequences are available in Additional file 3. The BAC clones were selected from the potato AFLP-fingerprint physical map [20], in which 202 *Eco*RI/*Mse*I-based AFLP markers from the RH ultradense genetic map [16] had anchored 141 BAC contigs containing 2441 BAC clones to Chr-5. With the addition of 9 AFLP markers that were identified by their sequence, in total 211 of the 668 available parental and bridge AFLP markers in the RH Chr-5 genetic map were linked to the MTP sequences. These AFLP anchor markers are located exclusively in the polymorphic chromosome regions and the seed BAC clones that carry these markers belong to either of the two opposite linkage phases of Chr-5, hereafter referred to haplotype {0} and haplotype {1}, respectively. For 91 tiling paths, sequencing was initiated in such AFLP-anchored seed BACs and was extended by selecting BACs of the same haplotype that overlapped based on AFLP-fingerprint or on BAC-end sequence (BES). An additional 16 Chr-5 tiling paths were identified through various other anchoring methods and for eleven of these the haplotype, {0} or {1}, could be indirectly inferred from the available physical map or sequence data (Additional file 1: Table S1). Five tiling paths remained without haplotype assignment (unphased). Of these, three (MTPs 178 {−}, 1382 {−}, and 1841 {−}) are taken to be from homozygous regions.

**Table 1:** **Statistics of sequenced chromosome 5 BACs**

**Number of BACs sequenced**	**573**
Total sequence length (bp)	70,278,808
Minimum BAC sequence length (bp)	14,260
Maximum BAC sequence length (bp)	216,437
Average BAC sequence length (bp)	122,651
N50 BAC sequence length (bp)	129,138

**Table 2:** **Chromosome 5 BAC tiling path sequence and AFLP marker statistics**

**Number of BAC tiling paths:**	**107**
Minimum length (BACs)	1
Maximum length (BACs)	25
Average length (BACs)	5.36
N50 length (BACs)	8
Minimum length (bp)	69,329
Maximum length (bp)	2,803,458
Average length (bp)	518,193
N50 length (bp)	748,401
BAC tiling paths with AFLP markers	91
Number of AFLP markers in tiling paths (a)	211
Average number of AFLP markers per tiling path	2.32
Minimum AFLP markers per tiling path	1
Maximum AFLP markers per tiling path	10
N50 number of AFLP markers per tiling path	3

(a) Includes 9 markers identified from the sequence data.

### BAC tiling path ordering

**Figure 1:**
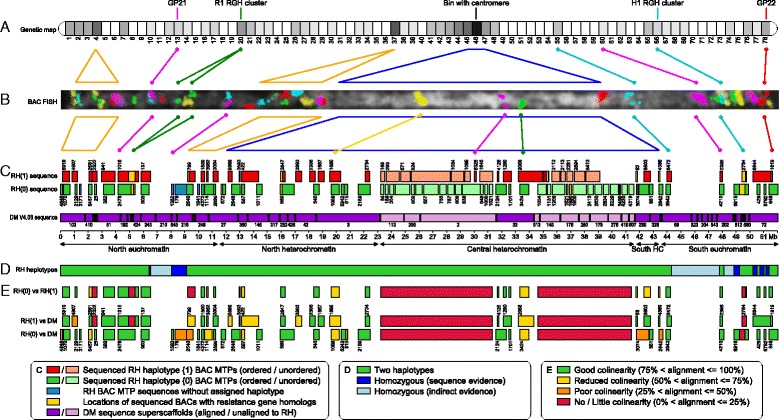
Alignment of the potato chromosome 5 genetic, cytogenetic and sequence maps. **A**. Chr-5 genetic map of genotype RH [16]. The map is divided in 78 bin segments of which the gray scale intensity corresponds to the number of AFLP markers per bin. Bin 46 has the highest marker density (174 markers) and contains the centromere. **B**. Digitally stretched cytogenetic map of RH pachytene Chr-5. Intense DAPI staining (white) marks the pericentromeric heterochromatin. Coloured foci mark the FISH positions of 35 BAC clones from the RH sequence tiling paths. For selected BAC clones, the connections are shown to the RH genetic (**A**) and sequence **(C)** maps. **C**. Alignment of the RH and DM genomic sequences. Positions of RH BAC tiling path sequences of haplotypes {0} and {1}, are shown as green and red blocks respectively, along the DM pseudomolecule sequence map (dark violet) of Chr-5. In the central heterochromatin, RH BAC MTPs of which the exact position is unknown are placed in arbitrary order and are shown in lighter colours. Likewise, DM sequence scaffolds without alignment to the RH sequences are shown in a lighter colour. The DM superscaffold sequences are marked only by their ID numbers, e.g. sequence block 103 at the start of Chr-5 is superscaffold PGSC0003DMB000000103 [18]. **D**. Model for the distribution of homozygous and polymorphic regions on RH Chr-5. **E**. Classification of sequence collinearity in overlap regions between RH and DM sequences. RH {0} vs RH {1} is the comparison between both sequence haplotypes in RH. RH {0) vs DM and RH {1} vs DM are comparisons of RH haplotype {0} and {1} sequences with the DM reference sequence.

The Chr-5 locations of the RH BAC MTP sequences are presented in Figure 1C, where haplotype {0} and haplotype {1} MTPs are shown as green and red blocks respectively, while MTPs without haplotype designation are shown in blue. The yellow overlay on these sequence blocks indicates the positions of BACs with disease resistance gene homologs [28]. The Chr-5 sequence map is divided into five regions: the north and south euchromatin, the north and south heterochromatin borders, and the central heterochromatin. The central heterochromatin harbours a large volume RH MTPs for which the precise location could not be determined, due to the lack of cross-over events between the anchoring markers and lack of mutual alignability (discussed in more detail below). These sequences were given arbitrary positions within the central heterochromatin, and are indicated by light green and light red boxes. The quantitative distribution of RH BAC sequences across the five regions of Chr-5 is shown in Table 3.

**Table 3:** **Quantitative distribution of the non-redundant BAC MTP sequences across regions of chromosome 5**

	**bp per chromosome region**					
**Sequence haplotype**	**North euchromatin**	**North heterochromatin**	**Central heterochromatin**	**South heterochromatin**	**South euchromatin**	**Totals**
RH homozygous	1,364,250				1,300,000 (a)	2,664,250
RH unknown			132,838			132,838
RH haplotype {0}	4,884,186	4,819,059	14,443,961	1,193,223	2,552,327	27,892,756
RH haplotype {1}	5,519,970	4,328,956	12,886,551	578,280	1,443,101	24,756,858
RH totals	11,768,406	9,148,015	27,463,350	1,771,503	5,295,428	55,446,702
DM sequence	9,973,565	12,734,159	17,195,885	1,791,234	7,825,315	49,520,158

(a) Estimated length of homozygous sequence incorporated in haplotype {0} MTP 6915 and haplotype {1} MTP 6844.

The primary source for ordering the BAC tiling paths has been the Chr-5 genetic map of genotype RH [16], which is integrated in the RH BAC physical map. This genetic map is composed of 78 bins, where each bin represents a chromosomal segment of which the markers are separated by one recombination event from markers in the adjacent bin (i.e. markers that are at *n* bins distance from each other, are separated by *n* recombination events). Bin segments contain varying densities of AFLP markers (Figure 1A). The highest marker density (black) is found in bin 46 [16,18]. The markers in this bin correspond to the heterochromatin region, where genetic recombination is absent and where the centromere is located [29]. Using the genetic map, the sequences were assigned to the north euchromatin, the heterochromatin, or the south euchromatin. Within the euchromatic regions, a partial ordering of MTPs was possible based on the marker bin numbers.

**Figure 2:**
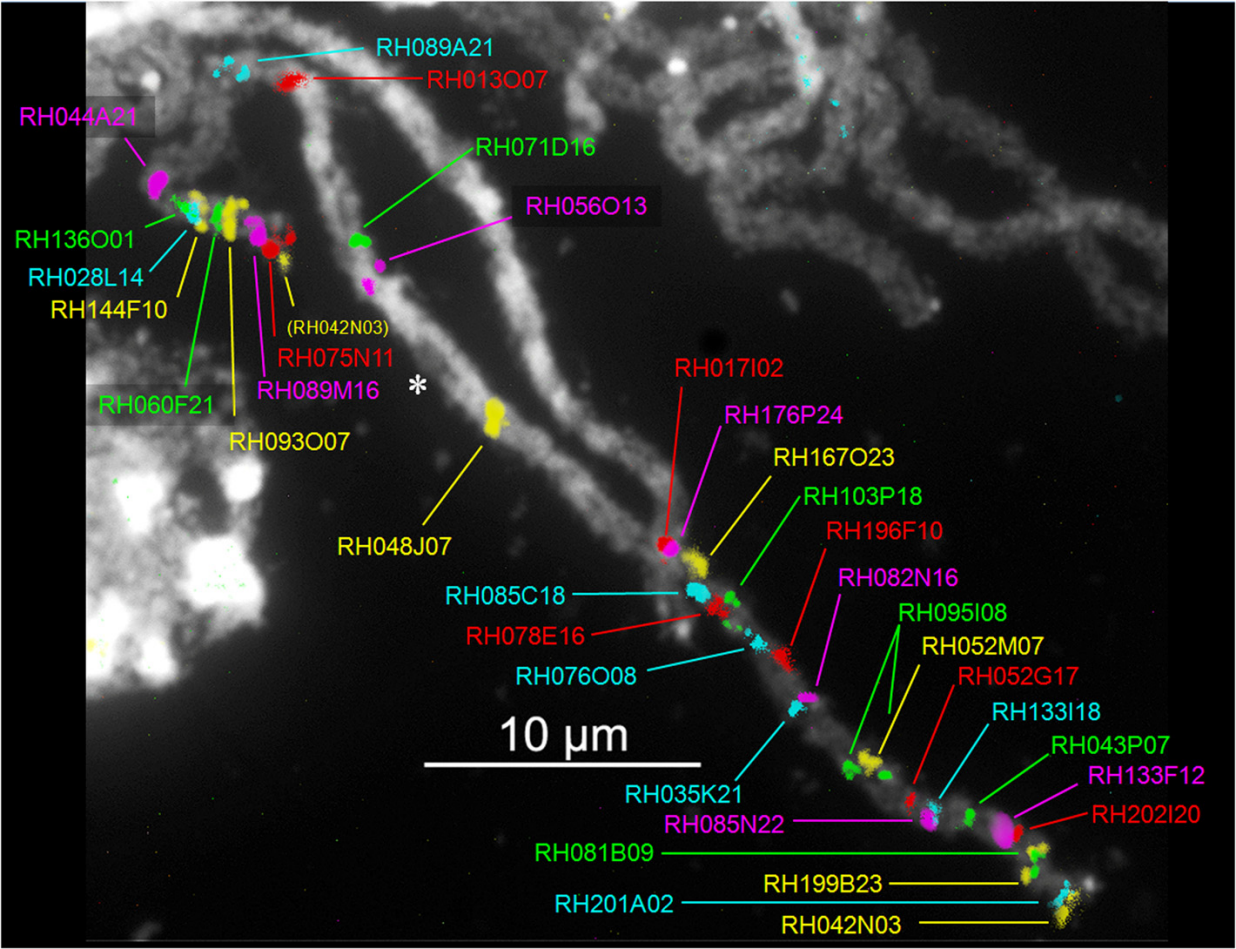
Detailed cytogenetic map of RH chromosome 5. This map was created by simultaneous fluorescence *in situ* hybridization of 35 BAC clones from the sequence MTPs to a pachytene chromosome spread. Intense DAPI staining of the condensed DNA of the pericentromeric heterochromatin is visible as white background to the colored BAC fluorescence signals. An asterisk marks the centromeric constriction. Subtelomeric BAC clone RH042N03 (yellow) produced a second fluorescence signal at the south end of Chr-5 (labelled in brackets). Eighty three percent of clone RH042N03 consists of the CL14 subtelomeric repeat [73,74] and this ectopic hybridisation signal most likely is caused by the presence of a similar subtelomeric repeat at the south terminus of Chr-5. Tiling path and marker data for the hybridized BACs are listed in Table 4.

BAC FISH to pachytene chromosomes was used for verification of several marker anchor points from the physical map, and for finalizing the ordering of the MTP sequences in the euchromatic regions. Thirty-five of the BAC clones that were used for these FISH experiments were combined in a single hybridisation with alternating labelling colours to produce a detailed cytogenetic map of RH Chr-5 (Figures 1B and 2; Table 4). In the euchromatic regions, the BAC hybridisations complemented the RH genetic map data and enabled a full ordering of the RH sequences. In total, 10 BACs from genetic bin 46 were also localized by FISH (Figures 1B and 2; Table 4). These hybridizations showed signal to locations across the entire heterochromatin and thus indicate that there has been no positional bias in the identification of BACs from this region.

**Table 4:** **Physical and genetic locations of BAC clones hybridized to potato chromosome 5**

**Chromosomal arm (a)**	**BAC clone**	**Figure**	**Color**	**Physical location (FL) (b)**	**MTP**	**Haplotype (e)**	**AFLP marker (number/name)**		**Genetic position (bin number) (f)**
5 L (EC)	RH042N03	1,2	Yellow	0.40 ± 0.06	4846	0	20179	EAAGMACC_272	4
5 L (EC)	RH201A02	1,2	Blue	1.14 ± 0.30	1070	0			(4)
5 L (EC)	RH081B09	1,2	Green	2.70 ± 0.42	2129	0	7241	EAACMCAG_149.7	4
5 L (EC)	RH199B23	1,2	Yellow	3.20 ± 0.66	3171	-			
5 L (EC)	RH202I20	1,2	Red	5.00 ± 0.42	6457	0	7202	EAGGMAGA_385	1-4
5 L (EC)	RH133F12	1,2	Magenta	5.88 ± 0.35	25	0	7235	EATCMCTC_236.2	4
5 L (EC)	RH043P07	1,2	Green	7.01 ± 0.33	941	1			(12)
5 L (EC)	RH133I18	1,2	Blue	8.52 ± 0.37	382	0	7260	PGA/MATG_101.5	12
5 L (EC)	RH085N22	1,2	Magenta	8.66 ± 0.76	1015	1	7265	EACAMACC_361.6	16
5 L (EC)	RH052G17	1,2	Red	10.07 ± 0.85	2478	0	20145	EAACMCTT_435H	16-17
5 L (EC)	RH095I08	1,2,3	Green/Blue	12.71 ± 1.15	1015	1	7280	EAACMAGG_167	21
5 L (EC)	RH052M07	1,2	Yellow	13.46 ± 1.13	903	0	7287	CAAGMCAT_174.6	26-27
5 L (EC)	RH035K21	1,2	Blue	16.64 ± 2.34	(c)				
5 L (EC)	RH082N16	1,2	Magenta	16.64 ± 2.34	1382	M			
5 L (EC)	RH196F10	1,2	Red	19.51 ± 1.25	178	M			
5 L (EC)	RH076O08	1,2	Blue	20.66 ± 1.32	799	1	20640	EAGAMACC_230	37
5 L (EC)	RH103P18	1,2	Green	21.76 ± 1.04	1841	M			
5 L (EC)	RH078E16	1,2	Red	23.02 ± 1.27	1800	1	7296	EAGGMACA_500	37
5 L (EC)	RH085C18	1,2	Blue	25.21 ± 2.02	1114	0			(37)
5 L (HC)	RH167O23	1,2	Yellow	24.76 ± 0.51	3004	1	11894	EATCMCAG_17H	37
5 L (HC)	RH176P24	1,2	Magenta	25.21 ± 2.02	672	0			(46–47)
5 L (HC)	RH017I02	1,2	Red	25.67 ± 2.33	672	0			(46–47)
5 L (HC)	RH048J07	1,2	Yellow	35.34 ± 2.78	1685	1	12427	EAGGMAAG_2H	47
Centromere		1,2,3	Asterisk	41.67					
5S (HC)	RH056O13	1,2	Magenta	45.11 ± 2.68	5325	1	11936	EACAMACC_270.9H	46-47
5S (HC)	RH071D16	1,2	Green	46.78 ± 2.45	3424	0	7590	EAGTMAGC_997	46
5S (HC)	RH102K09	3	Green	47.86 ± 2.38	1645	1	12129	EAGAMCCT_586.0H	46
5S (HC)	RH138C23	3	Magenta	47.93 ± 2.00	1645	1			(46–47)
5S (HC)	RH095M08	3	Yellow	51.94 ± 2.13	1054	0	7392	EAAGMCAT_15	45
5S (HC)	RH193O24	3	Red	52.03 ± 2.45	1054	0			(47)
5S (HC)	RH013O07	1,2	Red	57.28 ± 1.74	(d)				(46–47)
5S (EC)	RH089A21	1,2	Blue	60.68 ± 1.87	6472	1	12520	EACTMCTA_188.9H	55
5S (EC)	RH044A21	1,2	Magenta	68.57 ± 1.80	4210	0	7626	EAACMCTC_205	60
5S (EC)	RH136O01	1,2	Green	70.33 ± 1.47	6915	0	7629	EAAGMCTT_144.9	65
5S (EC)	RH028L14	1,2	Blue	71.12 ± 1.98	6915	0			
5S (EC)	RH144F10	1,2	Yellow	72.16 ± 1.14	6915	0	7631	CATAMCCA_322.3	66-69
5S (EC)	RH060F21	1,2	Green	73.51 ± 0.84	6844	M			
5S (EC)	RH093O07	1,2	Yellow	74.36 ± 1.14	429	0			
5S (EC)	RH089M16	1,2	Magenta	75.42 ± 1.17	6844	1	12569	EATCMCTC_27H	78
5S (EC)	RH075N11	1,2	Red	76.70 ± 0.93	648	0	7651	EAGTMCCA_208.5	78

(a) 5 L = long arm; 5S = short arm; EC = euchromatin; HC = heterochromatin.

(b) Physical location (= fraction length) was calculated as (S/T)binT, where S = the distance in μm from the FISH hybridization site to the north end of Chr-5, T = the total length of Chr-5 in micrometer, binT = the total bin value (78) of Chr-5.

(c) Unsequenced BAC clone aligning to DM superscaffold PGSC0003DMB000000210.

(d) Unsequenced BAC clone with marker GP188 and aligning to DM superscaffold PGSC0003DMB000000328.

(e) ‘-’ = haplotype undetermined; M = monomorphic region (i.e. no difference between haplotypes).

(f) Bin numbers in brackets are from AFLP anchors in adjacent BAC clones in the RH physical map. Bin values for AFLP markers can deviate slightly from the true bin value due to missing scores, e.g. in practice markers mapped to the bin 45–47 region all belong to the bin 46 segment of the genetic map.

Alignments of the RH sequences to DM Chr-5 were used for verification and further improvement of the RH MTP ordering. In the euchromatic regions, the sequence order given by the RH genetic map and RH FISH data was in full agreement with the order given by DM. The RH genetic map markers did not offer genetic resolution for sequence ordering in the heterochromatin. However, in DM the ordering of Chr-5 sequence superscaffolds does extend from the proximal euchromatic regions into the north and south heterochromatin borders. Twenty-seven more RH BAC tiling paths from genetic bin 46 were positioned in these north and south heterochromatin regions by alignment to the DM sequence.

FISH with BACs RH056O13 and RH071D16 directly positioned RH MTPs 5235 {1} and 3424 {0} from genetic bin 46 in the most central heterochromatin (Figures 1B, C and 2). Six RH tiling paths, including MTP 3424 {0}, were aligned to DM superscaffold 33. Through the FISH link of BAC RH071D16 (green in Figures 1B and 2) this ordered cluster of sequences could be placed near the middle of the heterochromatin.

A remaining set of 37 BAC MTPs with AFLP anchors to heterochromatic bin 46 of the RH genetic map could not be placed in a unified order, because of lack of sufficient and coherent sequence overlaps, either between the RH haplotypes or between RH and the corresponding DM superscaffolds of this region. These unaligned RH and DM sequence blocks are shown in lighter colours in Figure 1C. The order and orientation of the superscaffold sequences of DM in the central heterochromatin are only partly known and thus the sequence of this Chr-5 region remains to be finalised.

### Distribution of sequence polymorphism on RH chromosome 5

Although genotype RH is regarded to have a heterozygous genome, our sequencing of Chr-5 has identified homozygous regions as well. A model for the distribution of polymorphic and non-polymorphic regions on RH Chr-5 is given in Figure 1D.

In the north euchromatin, the first 6.3 Mb of sequence displayed two sequence haplotypes, which were distinguished and phased on the basis of the AFLP anchor markers and the sequence polymorphism between allelic BAC clones. In the RH physical map [20], BAC clones of opposite haplotype were often present in the same fingerprint contig. Therefore, close examination of BES overlaps was needed for this region in order to select extension BACs of the same haplotype from within the fingerprint contigs. Because of the generally close sequence collinearity between opposite haplotypes, this euchromatic region can be classified as heterozygous, i.e. as consisting of a continuum allelic sequences without much interruption by hemizygous segments.

In the interval from 6.3 to 9.3 Mb, the north euchromatin of RH is taken to be homozygous. A lack of AFLP anchor markers occurs in the genetic map in this interval, which is evidence of homozygosity between the two Chr-5 homologs. In the southern half of this interval, the homozygosity was confirmed in BAC MTPs 1382 {−} and 178 {−}. These MTPs were identified through BES links to sequenced tomato Chr-5 BACs. In alignments to the physical map, these MTP sequences displayed no sequence polymorphism towards the BES of their surrounding BACs. At the north boundary, the local disappearance of polymorphism is also visible in the flanking MTP 137 {1}, which shows a transition from homozygous into heterozygous sequence.

In the chromosome interval from 9.3 Mb to 44.3 Mb, which essentially spans the heterochromatin and its borders, the RH sequence has two different haplotypes. With the possible exception of a single-clone MTP 1841 {−}, at no point in this interval was there any evidence of homozygous sequences. In the physical map of this region [20], the fingerprint contigs were always completely separated by haplotype, which is evidence of strong sequence divergence between both chromosomal homologs. The sequence polymorphism in this interval is in part caused by heterozygosity of allelic sequences, but for a large part also due to hemizygous sequence segments that occur in one of the two chromosomal homologs. In the unaligned regions of the central heterochromatin, this hemizygosity is assumed to prevail. Both these latter aspects are discussed in more detail below.

In the chromosome interval from 44.3 to 47.8 Mb, which corresponds to the proximal half of the south euchromatin, the physical map had no AFLP markers for the identification of Chr-5 contigs and this unsequenced region is therefore likely to be homozygous in RH.

In the region from 47.8 Mb to 52 Mb, which corresponds to the distal part of the southern euchromatin, the RH sequence shows an alternation of polymorphic and homozygous regions. In the tiling path assembly, the homozygous sequence segments are merged into either MTP 6915 {0} or MTP 6844 {1}, each of which also include polymorphic sequence, having AFLP markers of haplotypes 0 and 1 respectively. MTP 6915 {0} has an alternate haplotype in MTP 2764 {1} at the position of the H1 resistance gene homologue (RGH) cluster, but becomes homozygous towards the north end [24]. The MTPs 429 {0} and 6792 {0} denote short intervals of allelic sequence that occur in parallel to MTP 6844 {1} at positions where the AFLP markers are located. At these two locations, the physical map fingerprints [20] of both haplotypes occurred within the same contig, and BES alignments were needed to separate allelic BAC clones for sequencing.

### Sequence collinearity and structural variation in the euchromatin

The structural divergence on Chr-5 was evaluated by dot-plot alignments between BAC MTPs of the two RH haplotypes and the DM reference genome. The overall results of these sequence comparisons are shown in Figure 1E for the RH versus RH and RH versus DM comparisons. Sequence overlap sections shown in green have a better than 75% collinearity within the sequence overlap interval. Aligned sequence segments with larger deviations in collinearity are marked with yellow, orange, or red colours. For regions with prominent deviations in sequence collinearity between the RH and DM haplotypes, the nature of these deviations is specified in more detail in Additional file 4: Table S3.

In general, the north and south euchromatic regions showed a near 100% sequence collinearity between the three haplotypes, with the occasional presence of inserts of up to several kb. An example is the 1750 kb stretch of continuous alignment between MTPs 648 {0} and 6844 {1} of RH versus DM in the south euchromatin (Additional file 5: Figure S1).

**Figure 3:**
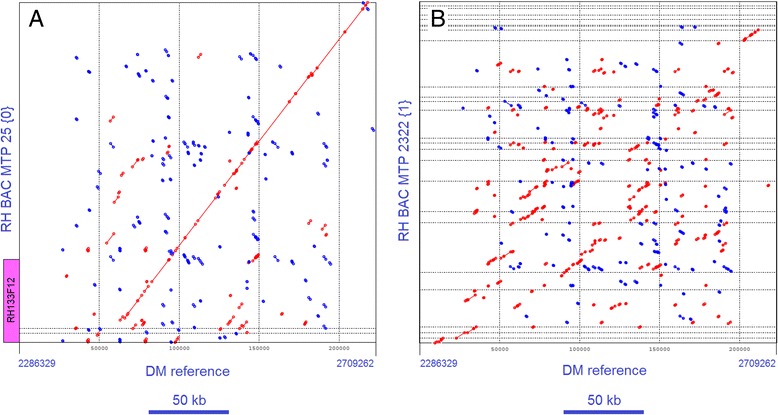
Dot plot alignment between RH and DM in a gene cluster in the north euchromatin of chromosome 5. **A**. Alignment of haplotype {0} BAC MTP 25 versus the DM reference sequence shows collinearity throughout a 150 kb region with sequence duplications. The position of BAC clone RH133F12 (magenta) is shown in MTP 25 {0}. This clone was used for in situ hybridization in Figures 1B and 2. **B**. At the same chromosome location, the RH haplotype {1} MTP 2322 has no collinearity to DM in the duplication region. This duplication region had caused problems in the DM genome assembly and the DM sequence used here for alignment is a concatenation of sequence fragments from four superscaffolds (410, 1176, 251 and 51), from which 150 kb of pseudomolecule gap spacing sequence has been removed.

In the euchromatin there is an abundance of sequence duplications where the colinearity of the two RH haplotypes is frequently reduced or completely lost (Additional file 4: Table S3). One example of this phenomenon is from the north euchromatin, illustrated in Figure 3A and B, that show the alignment of RH BAC MTPs 25 {0} and 2322 {1} to the DM reference sequence. These MTPs cover a 170 kb region and contain a mix of S-locus, F-box proteins and retrotransposon genes. While MTP 25 {0} is collinear with DM, the MTP 2322 {1} of the other haplotype is not. Another example is a large region with receptor kinase genes, which extends over approximately 200 kb in DM, and which is located in MTPs 382 {0} and 941 {1} of RH. The alignment of MTP 382 {0} to DM shows a partial loss of collinearity in this gene cluster (Additional file 5: Figure S2). Also the two other possible alignments, i.e. MTP 941 {1} to DM and MTP 382 {0} versus MTP 941 {1}, have a very large interruption of collinearity (data not shown), resulting in three different haplotypes for this gene cluster.

Clusters of disease resistance genes and their homologs are known to display high sequence diversity, which can lead to local deviations from sequence collinearity between chromosome haplotypes [30]. The R1 late blight resistance gene homolog (RGH) cluster is situated in the north euchromatin of Chr-5, and is marked by BAC clone RH095I08 in the cytogenetic map (Figure 2). Within genotype RH, two distinct haplotypes are found for this R1 cluster, in respectively BAC MTP 2478 {0} and BAC MTP 1015 {1}. With sizes of respectively 185 kb and 385 kb, these R1 clusters have a considerable difference in length, and dot-plot alignment reveals that they have no mutual sequence collinearity apart from the conserved domains shared by the paralogous *R*-genes (Additional file 5: Figure S3A). When the two R1 haplotypes of RH are aligned to DM, the shorter haplotype {0} cluster in MTP 2478 is collinear with DM, while the longer haplotype {1} cluster in MTP 1015 is not (Figure 1E; Additional file 5: Figures S3B, S3C). In a further comparison, no sequence collinearity was found between either of the two susceptible R1 cluster haplotypes from RH and two previously published susceptible and resistant R1 cluster haplotypes in potato [30].

The H1 RGH cluster on the south euchromatic arm is located between BACs RH028L14 and RH144F10 in the cytogenetic map (Figure 2) and has its sequences in RH MTPs 6915 {0} and 2764 {1}. It was previously shown that no sequence collinearity exists between the two haplotypes of this cluster in RH (susceptible) and the haplotype conferring cyst-nematode resistance from diploid clone SH83-92-488 [24]. Comparison of these three H1-region clusters to the DM reference genome (susceptible) again revealed no sequence collinearity (data not shown).

The alignment of RH MTP 1015 {1} to the DM reference sequence shows both a small and a large F-box gene cluster, situated at opposite ends of the R1 RGH cluster, where collinearity is broken (Figure S3B). In RH MTP 2478 {0}, the small F-box gene cluster does show collinearity with DM, while the large F-box gene cluster could not be evaluated because it was not sequenced (Additional file 5: Figure S3C). More examples of collinearity loss in gene duplication regions of the euchromatin are listed in Additional file 4: Table S3.

**Figure 4:**
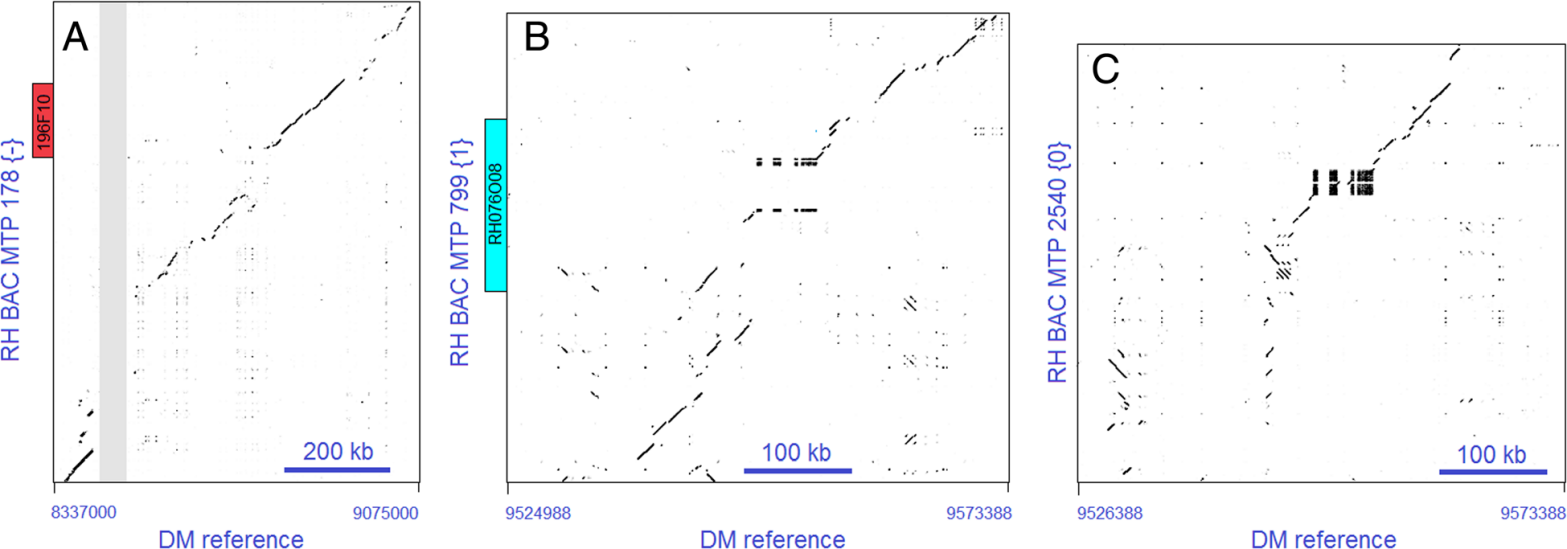
Regions with substantial loss of sequence collinearity in the proximal part of the north euchromatin of chromosome 5. **A**. Alignment of RH BAC MTP 178 {−} versus the DM reference sequence. The grey zone marks 50 kb of spacer sequence that fills a gap of unknown length between superscaffolds 540 and 216 in the DM pseudomolecule sequence. The position of BAC clone RH196F10 is shown in MTP 178 {−}. This clone was used for in situ hybridization in Figures 1B and 2. **B**. Alignment of RH haplotype {1} BAC MTP 799 versus the DM reference sequence. The position of BAC clone RH076O08 (blue) is shown in MTP 799 {1}. This clone was used for in situ hybridization in Figures 1B and 2. **C**. RH haplotype {0} BAC MTP 2540 versus the same DM region as in **(B)**. The dark boxes in alignment **(C)**, which are also partly visible in alignment **(B)**, are caused by a 180 bp sized tandem repeat.

Extensive losses and interruptions in sequence collinearity occurred in the proximal part of the north euchromatin. MTP 178 {−} contains 925 kb of sequence from the homozygous region in RH, of which the alignment to the DM reference genome is poor or broken (Figure 4A). In MTPs 799 {1} and 2540 {0}, a similarly strong degradation of collinearity is observed, both in the alignment to DM (Figure 4B, C) and in the mutual alignment of these RH MTPs (data not shown). In addition, at the heterochromatin border, the haplotype {0} MTP 1114 contains an exceptionally large insert of 315 kb that is not present in RH haplotype {1} MTP 3962 or in DM.

### Sequence collinearity and structural variation in the heterochromatin

The generally tight sequence collinearity between the RH and DM haplotypes in the euchromatin degrades as the sequence moves from the euchromatin into the heterochromatin borders. This is manifested by the continuous presence of indels, ranging from a few kb up to several tens of kb, which cause fragmentation of the alignment patterns (Additional file 5: Figure S4). However, even with these fragmentations, many of the heterochromatic sequence alignments remain at 75% collinearity, which is still classified as good alignment (green) in Figure 1E.

**Figure 5:**
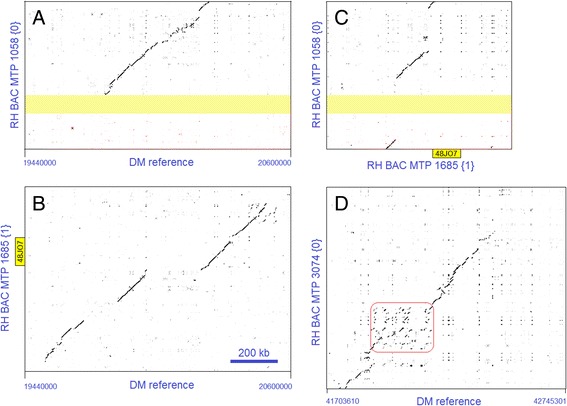
Examples of large breaks in sequence collinearity in the heterochromatin borders. **A**. Alignment of RH haplotype {0} MTP 1058 to the DM reference genome. The start of MTP 1058 {0} is missing in DM. The yellow band indicates an unsequenced region in MTP 1058 {0}, estimated to be 80 kb in length. **B**. Alignment of RH haplotype {1} MTP 1685 to the same section of the DM reference genome. Alignment gaps of 145 kb and 164 kb to DM are found at different positions compared to **(A)**. The position of BAC clone RH048J07 (yellow) is shown in MTP 1685 {1}. This clone was used for in situ hybridization in Figures 1B and 2. **C**. Alignment of the two RH haplotypes shows two large inserts of 188 kb and approximately 266 kb in MTP 1058 {0}. The position of BAC clone RH048J07 (yellow) is shown in MTP 1685 {1}. This clone was used for in situ hybridization in Figures 1B and 2. **D**. The terminal 270 kb of RH MTP 3074 {0} from the south arm has no overlap with the DM sequence. The remaining part of MTP 3074 {0} has a very fragmented collinearity with DM, which disappears in the boxed area. This box marks a sequence duplication region that contains - among others - five DNA repair helicase genes. Scale bar 200 kb applies to all figure panels.

Severe breaks in collinearity, involving sequence lengths from 91 (MTP 860 {0}) to 270 kb (MTP 3074 {0}) were found at seven locations in the north and the south heterochromatin borders (Additional file 2: Table S2). As an example, Figure 5A, B, C show alignments from the north heterochromatin of the overlapping RH BAC tiling paths 1058 and 1685 in relation to the DM sequence. Each of the pair-wise haplotype comparisons gives one or two breaks of at least 145 kb in the alignment. Remarkably, RH MTP 1058 {0} gives a better alignment to DM than to RH MTP 1685 {1}. Another example, from the south heterochromatin border, is given in Figure 5D, where the terminal 270 kb of RH MTP 3074 {0} has no match to the DM sequence at this same physical location. This same figure shows the loss of collinearity at a cluster of DNA repair helicase genes.

In the central heterochromatin, DM superscaffold 33, located at position 32–34 Mb (Figure 1C) gave highly contrasting qualities of alignment to its matching RH sequences. Within a 2 Mb distance on this DM superscaffold, an accurate sequence collinearity was found with RH BAC MTPs 1280 {1}, 1701 {0}, 2124 {0} and 4125 {1}, whereas RH MTPs 3424 {0} and 2058 {1} presented a fragmented alignment with duplication regions (Additional file 5: Figure S5).

Dot-plot comparisons between the remaining central heterochromatic RH MTPs and DM superscaffolds of unknown chromosome position (light colours in Figure 1C) revealed only limited amounts of sequence overlap, with a highest overall value of 36 percent overlap between the RH haplotype {0} MTPs and the DM sequence scaffolds (Table 5; Additional file 5: Figure S6). The overlapping sequences typically consisted of isolated segments of 50 to 250 kb length. Incongruences were encountered when trying to align haplotypes to each other. For example, RH MTP 838 {0} showed five alignment segments in two DM superscaffolds, of which the distant positions and partially reversed orientations were difficult to reconcile with general sequence collinearity between these haplotypes. Because of these severely broken and sometimes conflicting alignments, no further efforts could be made to find a unified order for these remaining central heterochromatic RH and DM sequences.

**Table 5:** **Quantification of overlaps between unaligned sequences in the central heterochromatin of chromosome 5**

**Haplotype sequence**	**Number of sequences (MTPs or superscaffolds)**	**Total length (kb)**	**Overlap between haplotypes in kb (above diagonal) and in percentage (below diagonal)**		
			**RH haplotype {0} MTPs**	**RH haplotype {1} MTPs**	**DM superscaffolds**
RH haplotype {0} MTPs	24	12764	--------	2550	4608
RH haplotype {1} MTPs	13	10004	25.5 (a)	--------	2104
DM superscaffolds	10	15192	36.0 (b)	21.0 (b)	--------

(a) Percent phase 1 sequence recovered in phase 0 sequence.

(b) Percent RH sequence recovered in DM.

### FISH positioning of co-aligning sequences in the central heterochromatin

**Figure 6:**
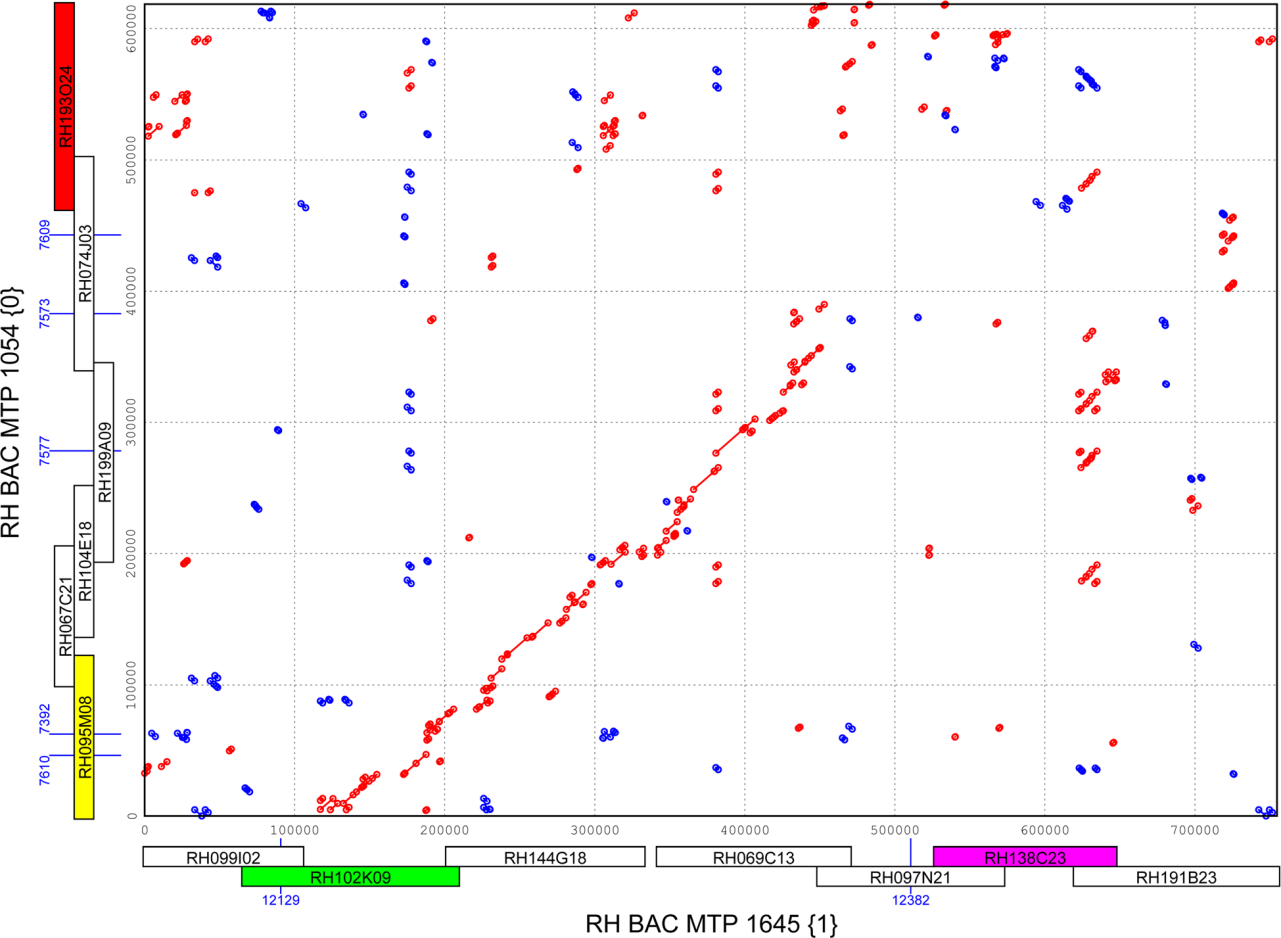
Dot-plot alignment of putative allelic RH BAC tiling paths from the central heterochromatin. RH MTP 1054 of haplotype {0} has fragmented collinearity with RH MTP 1645 of haplotype {1} over a length of 350 kb of sequence, suggesting that these MTPs are allelic. The BAC clones that make up these tiling paths are depicted on the axes. Four BAC clones (colored) have been selected for cytogenetic mapping by FISH in order to verify this possible allelism. The blue ID numbers on the axes correspond to AFLP markers from bin 46 of the genetic map, which anchor these sequenced BACs to the corresponding haplotypes of RH Chr-5.

The limited and conflicting sequence overlaps between the RH and DM haplotypes in the central heterochromatin raised doubts as to whether the segments of sequence that do align are allelic sequences coming from identical physical positions on the chromosome. This issue was addressed using BAC FISH to pachytene chromosomes, using clones from RH MTPs 1054 {0} and 1645 {1}, of which the exact chromosome positions were unknown. Tiling paths 1054 and 1645 are genetically anchored by multiple AFLP markers in genetic bin 46, to the phase 0 and phase 1 haplotypes of Chr-5, respectively. Their alignment has a 350 kb segment of collinear sequence, followed by 270 kb of non-collinear sequence (Figure 6).

**Figure 7:**
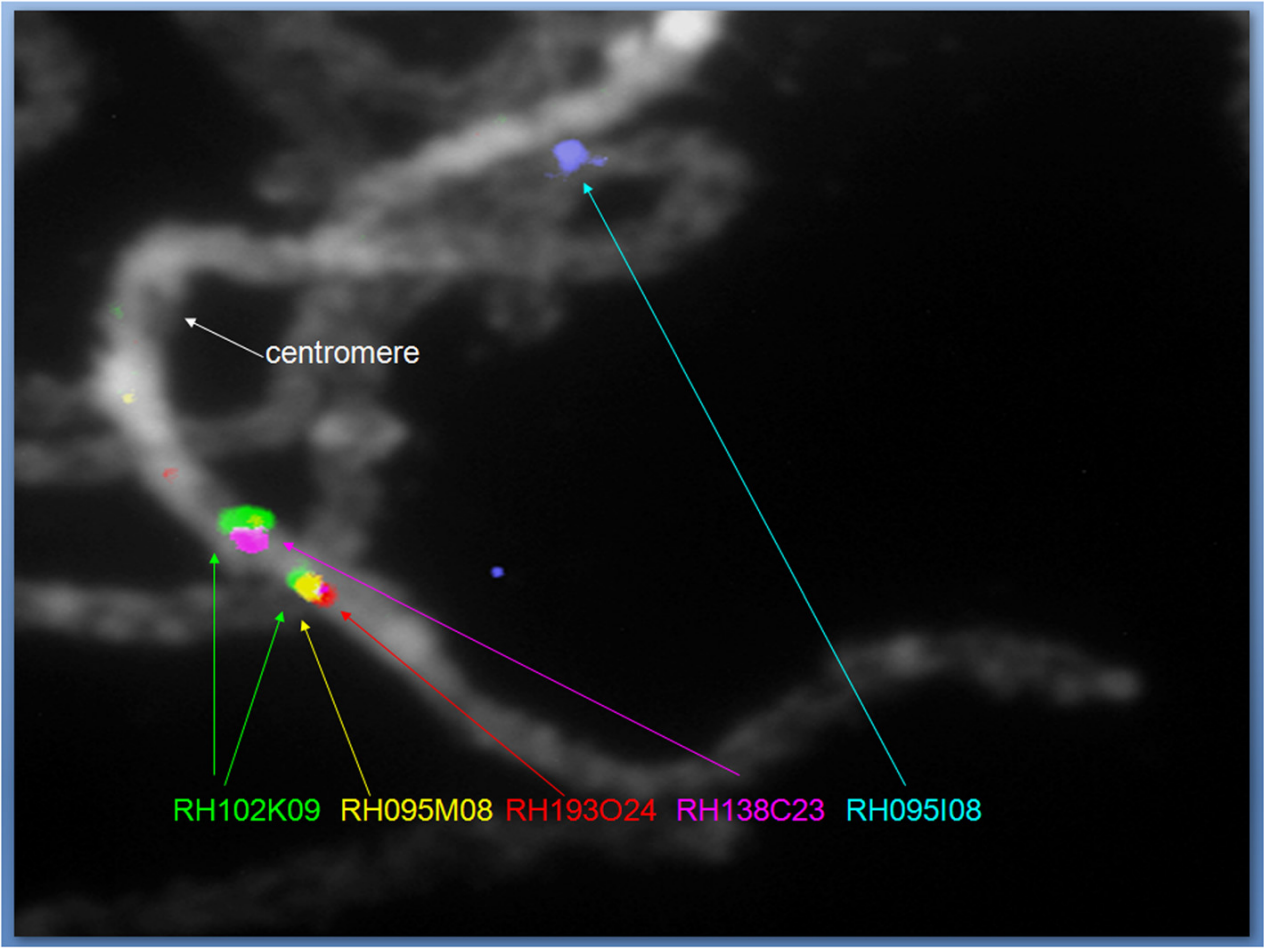
Pachytene BAC FISH verification of putative allelism of MTPs 1054 and 1654 from the central heterochromatin of chromosome 5. BAC clones RH095M08 and RH193O24 from the haplotype {0} MTP 1054 map to a cytogenetic position that is different from the location of clones RH102K09 and RH138C23 from haplotype {1} MTP 1645. This means that these two MTPs are not allelic, despite their partial sequence overlap. Clone RH102K09 gives an additional signal at MTP 1054 {0}, which is presumably cross hybridisation to sequence from the overlap area in MTP 1054 {0} that is not covered by hybridisation with the shorter clone RH095M08. Clone RH095I08 marks the north euchromatic arm of Chr-5.

Hybridizations were carried out with two clones from the area of sequence overlap (RH095M08 and RH102K09) and two clones from the non-overlapping area (RH193O24 and RH138C23) of these MTPs. Although the hybridizations showed that the two MTPs are indeed located in the central heterochromatin, they were found to occupy different cytogenetic positions (Figure 7). The conclusion therefore is that these two MTPs with partially overlapping sequence are not allelic at all, in the sense that their sequences are not juxtaposed on the paired homologous chromosomes during the pachytene phase. This result confirms the suspicion that partially co-aligning sequence blocks in the central heterochromatin need not come from the same chromosome location, and that errors in the ordering and positioning of sequences can be made when trying to use these partial overlaps.

### Correlation between genetic, cytogenetic and sequence maps

Figure 1 illustrates the distance relationships between the genetic, cytogenetic and sequence-based Chr-5 maps of genotype RH. The heterochromatin region is conspicuous by occupying no more distance on the genetic map than a single marker bin, but expanding to 40% of the chromosome’s length in the cytogenetic map (Figure 1B, blue triangle). The condensed nature of the pachytene heterochromatin (bright white) is revealed when this is aligned to the sequence map (Figure 1C, blue trapezium), where it is expanded further to the interval from 11 to 45.5 Mb, which corresponds to 65% of the sequence length of Chr-5. In the RH genetic map, the regions with genetic recombination activity run from bin 1–45 on the north arm, and from bin 47 to 78 on the south arm. These regions where cross-overs are observed correspond to the north and south euchromatin, which in the sequence map have lengths of respectively 11.5 Mb and 8.5 Mb only. Cross-over events are thus confined to approximately 38% of the 52 Mb total chromosome length.

At the top of the genetic map of Chr-5, bins 1–3 were found to have no clear sequence equivalent. In the sequence MTPs, markers from bins 1–3 become positioned between markers from bin 4. This could be an artefact caused by the method of linkage map construction [16], where flanking markers were used to correct putative data errors. Markers located at the most distal positions of the map do not have flanking markers anymore to allow rigorous inspection of marker order. Alternatively, since bin 1 is defined exclusively by six markers of the *Pst*I/*Mse*I enzyme combination (and does not contain AFLP markers from the other two enzyme combinations in the ultradense genetic map), bin 1 may be an artefact caused by a systematic error in the dataset of this enzyme combination.

In addition to heterochromatic bin 46, also euchromatic bins 4 and 37 of the Chr-5 genetic map have an elevated marker density. The density plot values used in Figure 1A are 19, 23 and 174 parental markers for bins 4, 37 and 46, respectively. The elevated marker densities in bin 4 and 37 are best explained by an increased local sequence polymorphism, as can be seen in Figure 1E.

## Discussion

### Haplotype-specific genome sequencing reveals a high level of sequence diversity

Potato is the first plant species in which long-distance haplotype-specific sequence assemblies have been produced from a heterozygous genome [2]. For the diploid clone RH, our BAC-by-BAC sequencing approach has resolved 55.4 Mb of non-redundant genomic sequence from Chr-5. Our sequencing effort exploited the capacity of the RH physical map [20] to separate BAC clones from the two Chr-5 homologues, based on AFLP markers, resulting in 52.6 Mb of haplotype-specific sequence. Also, the abundance of AFLP markers in the pericentromeric heterochromatin of RH Chr-5 has given us the unique opportunity to identify and sequence clones from this relatively unexplored genomic region, in which other types of molecular markers often give very limited coverage for anchoring.

With the two RH Chr-5 BAC assemblies, and the available monoploid DM reference genome, we were able to compare three independently constructed sequence haplotypes on a chromosome-wide scale. This comparison has revealed an unprecedented level of sequence variation between homologues of a plant chromosome, which sheds new light on plant genome biology, both from a sequence-based perspective and from a cytogenetic standpoint.

### Intraspecific genome diversity

The basic assumption when aligning sequences from homologous chromosomes of the same species is that these will be essentially collinear throughout their full length [31]. This assumption, however, is being challenged since the discovery that significant structural variations are possible that disrupt sequence collinearity [31,32]. These structural variants can exist as differences in gene copy number, but can also be caused by larger insertion and deletion events [32]. Structural differences have been reported mainly between varieties of the same plant species [33], but also between homologous chromosomes in heterozygous genomes [4,14]. Their occurrence has led to the concept of a pan genome, which is composed of a core genome of ever-present genomic elements and a dispensable genome that is partially shared between individuals [33,34].

Disease resistance gene clusters were one of the first and best-studied regions of structural variation in plants, where birth-and-death evolution takes place through unequal recombination, leading to gene duplications and diversifications that are beneficial in the co-evolution with pathogens [35,36]. Furthermore, in maize the insertion of long-terminal-repeat transposons was recognized at an early stage as a main cause of structural variation between inbred lines [31].

The recent progress in plant genome sequencing has enabled examination of structural variation at a genome-wide scale. In a whole genome assembly of the heterozygous grape variety Pinot Noir, it was estimated from chromosome-specific gaps of on average 49 bp, and from haplotype-specific assembly contigs of on average 3000 bp, that homologous chromosomes differ on average by 11.2% of their DNA content [4]. Comparative genomic hybridizations and re-sequencing efforts between genotypes of the same species have focused on the detection of copy number variation (CNV) and presence-absence variation (PAV) in genes and non-coding regions of several plant species [33]. In maize, rice and soybean the affected regions generally reached sizes up to a few tens of kb [37-39], but extreme values of 180 kb and 2.6 Mb have also been found for single PAV segments in rice and maize [37,38].

Previous research in potato has already pointed at the structural variation that can be expected in the genome of this crop species. For the approximately 10% of the diploid RH genome that is currently sequenced, alignment to the DM reference genome showed 275 RH genes to be absent in DM, while 29 genes in these compared regions were DM specific [2]. In addition, in tetraploid potato, pachytene BAC FISH has demonstrated a frequent occurrence of 137–145 kb segments in the euchromatic regions of chromosome 6 that show CNV within and between cultivars [14].

### Structural variation on potato chromosome 5

In the present paper, we have used dot-plot alignments to evaluate the continuity and interruptions in sequence collinearity on Chr-5 between the RH BAC tiling path sequences and the DM reference sequence. In this analysis we did not examine copy number variation at the level of individual genes or specific genetic elements, but instead focused on documenting the larger elements of structural variation that cover approximately 20 kb and more.

Structural diversity was examined in the euchromatin and in the heterochromatin borders of Chr-5, where 32.7 Mb of the RH sequence could be unambiguously positioned on the DM reference genome. In the most northern and southern euchromatic regions, the overall sequence collinearity was nearly 100%. Interruptions were limited to eight gene clusters, of which three contain disease resistance genes, two contain F-box genes and the other three had flavonol sulfotransferases, receptor kinases and MADS-box genes respectively. These collinearity interruptions indicate that at non-disease resistance gene clusters, birth-and-death evolution of multigene families also takes place [40]. The cluster organisation of F-box genes and receptor kinases in potato is consistent with findings in *Arabidopsis*, where these gene families are highly abundant and are also organised in tandem repeat clusters [41,42]. Closer to and within the heterochromatin borders, the structural variation changed in character, often consisting of one-sided inserts, with sizes of up to 315 kb. Alternatively, juxtaposed sequence segments were found with limited or no sequence similarity and of unequal length. In these cases, the sequence segments causing the variation can be very long, for example 270 kb of sequence in RH MTP 3074 {0} has no match to either the other RH haplotype or DM. We found these large structural variations to be quite abundant in the proximal euchromatin and in the heterochromatin border regions: here, the frequency of regions with less than 75% of sequence collinearity was 7 out of 14 for the comparisons between the RH tiling paths, and 13 out of 24 for the comparisons between the RH tiling paths and DM.

### Complete loss of collinearity in the pericentromeric region

We have revealed an extreme sequence divergence in the pericentromeric region of Chr-5. Although within the central heterochromatin a limited set of RH sequence blocks could still be aligned to DM superscaffold 33 and positioned by a single FISH anchor, a credible ordering of the remaining large volume of RH and DM sequence blocks in this region was not possible. The very limited, fragmented, and sometimes contradicting sequence overlaps prevented chaining of these RH and DM blocks into larger scaffolds for gap closure in the pericentromeric heterochromatin. Moreover, the validity of using the sparse sequence overlaps for mutual ordering of the central heterochromatic sequence blocks was drawn into question, when cytogenetic mapping demonstrated that two RH sequence tiling paths of opposite haplotype that carried a closely related sequence segment were actually from different physical locations on Chr-5.

The poor alignability in the central heterochromatin cannot be dismissed on the grounds that most of this region would still be unsequenced. With a length of 52.1 Mb, the current DM Chr-5 pseudomolecule assembly [18] is close to two cytologically determined size estimates of 56.13 and 60.95 Mb respectively [22]. This means that the 17.2 Mb of DM sequence that is placed in the central heterochromatin must be nearly complete for this region, and that the coverage by RH sequences of both haplotypes is also close to complete (Table 3). If even a modest level of collinearity had existed in the central heterochromatin, this should have been apparent from the available sequences.

From these combined observations it is concluded that the three central heterochromatic sequence haplotypes of Chr-5 in RH and DM must be extremely dissimilar over physical lengths exceeding 10 Mb. A working model for this region is to regard the three central heterochromatin haplotypes as completely independent sequences, in which sections of related sequence can be identified, but which do not necessarily point to identical physical positions on the chromosome.

This heterochromatic sequence divergence is of a magnitude that matches inter-genomic comparisons between related plant species [43]. The heterochromatic sequence scaffolds of DM Chr-5 that could not be ordered in parallel to the RH sequences cover 15.2 Mb (29.1%) of the total chromosome length. After subtraction of the scattered syntenic regions between DM and RH, we crudely estimate that respectively 18.6% and 22.7% of the full DM Chr-5 sequence is different from the RH haplotype {0} and haplotype {1} in the central heterochromatin (Table 5). These values are of the same scale as the 22.2% difference in sequence alignability between *A. thaliana* and *A. lyrata* [43].

The tomato genome is highly congruent with potato, having an identical chromosome number, near-identical genome size and the same overall chromosome architecture, featuring euchromatic chromosome arms that encompass a pericentromeric heterochromatin [44]. The layout of the tomato genome sequence has been compared with that of potato using dot-plot sequence alignments, in order to establish inversion break points [18]. These comparisons also showed that the sequence of tomato Chr-5 aligns with the DM reference genome from the euchromatin into the heterochromatin borders over the same distance where the RH sequences were aligned to DM. However, in the central heterochromatin region, where the RH haplotypes differ from DM, the tomato sequence also loses all similarity to DM. Thus, the heterochromatic sequence divergence on potato Chr-5 is of the same magnitude as the sequence difference between tomato and potato in this region.

It remains to be determined whether the three heterochromatin haplotypes of chromosomes 5 are an exceptional situation, or whether such diversity also exists on the other chromosomes of cultivated potato. The limited amount of genomic sequence that is currently available for the other chromosomes of RH precludes such an analysis by direct sequence comparison to DM. However, in the process of ordering the DM reference sequences [18], we have also had a close look at sequence tag alignments of the complete RH BAC physical map to the DM reference chromosomes. These unpublished results indicate that within genotype RH, Chr-5 indeed stands out by giving a poor, gapped RH BAC coverage of the DM heterochromatin. For the other RH chromosomes, the BAC clones show a much better coverage of the heterochromatic regions in DM, indicating that one or both haplotypes in RH will be more similar to DM.

### Pericentromeric sequence divergence does not preclude chromosome pairing during pachytene

The precise mechanisms for recognition and alignment of homologous chromosomes during meiosis in plants are still poorly understood [45]. During the pachytene stage of meiosis, the homologous chromosomes occur aligned side-by-side in the synaptonemal complex [46]. It is generally assumed that the DNA sequence itself is important for homology recognition [47] and double strand break formation and recombination have been implicated in chromosome pairing [45]. However, in many organisms homologous pairing has been found to be independent of recombination [45], as must be partially the case also in potato, where genetic recombination is repressed in the heterochromatin [21,29]. Alternatively, it has been proposed that the rough alignment of the synaptonemal complex depends on bringing together key allelic transcription units [48].

With the highly divergent heterochromatin haplotypes on RH Chr-5, it can be excluded that such recombination-dependent or sequence-driven mechanisms play a role in chromosome pairing in this pericentromeric region. Possibly, synaptonemal alignment is initiated in the collinear parts of the RH euchromatin, but then proceeds into the heterochromatin using a different recognition mechanism.

From the case of RH Chr-5, we conclude that potato chromosomes tolerate up to 15 Mb of sequence in their heterochromatin that lacks long-distance, side-by-side sequence collinearity with the heterochromatin in the homologous chromosomes. It is only at the euchromatic chromosome ends that homologous sequences are consistently co-aligned during meiosis and that crossovers occur. This leaves the heterochromatin as a major, multi megabase-sized sequence haploblock that may be passed on between generations without modification, and without the need of having to fulfil any requirements of sequence collinearity. In this respect, genomic heterochromatin sequences may offer a new and powerful lead for tracking pedigree and phylogenetic relationships in potato and other solanaceous species.

### Origin of pericentromeric sequence divergence

The notion that three highly dissimilar pericentromeric haplo-blocks were found in the heterochromatin of potato Chr-5, raises questions on their origin. In its immediate pedigree, the sequenced genotype RH descends for 75% from diploid lines that originate from tetraploid *S. tuberosum* group Tuberosum cultivars, while 25% of its genetic background comes from two diploid *S. tuberosum* Group Phureja accessions [2,49]. The DM reference genotype belongs to Group Phureja, and because both Chr-5 heterochromatin haplotypes in RH differ from DM, it is likely that RH inherited its haplotypes via Group Tuberosum ancestry.

The observed heterochromatic sequence divergence between the potato Chr-5 haplotypes is most likely caused by proliferation of transposable elements and by deletions from illegitimate recombination events between these elements, which are considered the major driving factors of plant genome evolution [50,51]. In a comparison between sorghum and rice, interspecific genome micro-synteny was virtually excluded from the heterochromatin and pericentromeric regions, indicating that activity of mobile elements is causing breakdown of collinearity and sequence divergence especially in these regions [52,53].

Three explanations are possible for the occurrence of unrelated heterochromatin haplotypes on potato Chr-5. Firstly, Chr-5 has been a location for introgression breeding of nematode and late blight disease resistance genes from wild potato species such as *Solanum demissum*, *S. vernei*, *S. acaule*, and *S. tuberosum* Group Andigena [24,25,54,55]. This explanation, however, can be excluded, as RH does not carry such resistance genes, nor do the parents or (great)grandparents in its pedigree [2].

Secondly, it has been proposed that cultivated tetraploid potato has an allopolyploid origin that combines genomes from two or more diploid ancestral species [56,57]. The two RH heterochromatin haplotypes may thus be indicative of this putative hybrid origin of cultivated potato. Such allopolyploidy would then still have to allow for tetrasomic chromosome pairing in cultivated potato. This may be explained by assuming that key chromosomal areas, such as the euchromatic arms, still have sufficient homology or other recognition mechanisms for autotypic chromosome pairing and recombination in a chromosome set of otherwise allotypic origin.

Thirdly, the suppressed genetic recombination may be a factor involved in the heterochromatic sequence divergence on potato chromosome 5. Suppression of recombination in the pericentromeric region has been documented also for the other potato chromosomes [16,21] and is presumably associated with accumulation of repeated sequences and the formation of modified chromatin structure in these regions [58,59]. In the euchromatic arms of chromosome 5, meiotic recombination ensures sequence homogenisation and concerted evolution of low complexity DNA by gene conversion [60]. By contrast, the pericentromeric regions are in reproductive isolation from each other due to their suppressed recombination. These large haploblocks can thus follow an uninterrupted route towards sequence divergence. A question remains, however, whether sequence evolution in *S. tuberosum* can have progressed fast enough to produce the level of divergence that is currently seen on Chr-5.

## Conclusions

We have generated *de novo* sequence data for two haplotypes of Chr-5 in the diploid potato clone RH and have compared these sequences to each other and to the third haplotype of Chr-5 in the DM potato reference genome. This comparison between three independent sequence assemblies has allowed an unbiased evaluation of large sized structural variation on Chr-5. A full spectrum of sequence divergences between haplotypes was encountered, ranging from homozygous regions, via well-aligned heterozygous regions, to interruptions by inserts well in excess of 100 kb, and regions where sequence collinearity between haplotypes disappeared. The most notable absence of sequence homology was found in the central heterochromatin, where co-alignment of RH and DM haplotypes grossly failed.

Our results on Chr-5 confirm the reports from BAC FISH on chromosome 6 that structural variations covering large sequence segments are present in the euchromatin of the potato genome [14], and we now show that such variations are also abundant in the heterochromatin. The sizes of the structural variations on potato Chr-5 clearly exceed the average values reported previously for other plant genomes, such as maize, rice and soybean [37-39]. The discovery of quite unrelated heterochromatic sequences within the same species is a novelty in plant genome analysis, and shows that in potato homologous chromosomes do not require sequence homology in this region in order to complete meiotic pairing.

The consequence of these structural variations is that the current DM potato reference genome can only support the resequencing of more potato genotypes at euchromatic regions, and that additional *de novo* assemblies are preferred for truly resolving all sequence variation in potato. At present *de novo* genome sequencing of tetraploid potato cultivars is still a challenge, however, the development of novel approaches, such as long-read single molecule sequencing and optical mapping, may change this outlook and enable the complete dissection of structural variation in heterozygous polyploid genomes in the near future [61,62].

## Methods

### BAC sequencing

BACs for sequencing were taken from the RHPOTKEY BAC library [20]. A total of 527 BACs were sequenced individually by the dye terminator (Sanger) method, producing paired end sequences from 2 kb cloned fragments at approximately 6x coverage, either at GATC Biotech AG (Konstanz, Germany) or at Macrogen (Seoul, South Korea). The BAC sequence reads were assembled with the Staden Package [63]. The Sanger sequenced BACs were on average 121 kb in length, with on average 10.0 assembled sequence contigs per clone. A selection of 70 clones were sequenced with the Roche 454 GS FLX pyrosequencing procedure, either at Roche Applied Science (Almere, The Netherlands) or at the Greenomics sequencing facility of Wageningen UR (Wageningen, The Netherlands). For 454 sequencing, sheared DNA fragments of 2 kb length were prepared individually per BAC clone and were labelled with one of twelve MID barcode adapters according to the manufacturer’s protocol (Roche Applied Science, Almere, The Netherlands). Barcoded BAC DNAs were combined in DNA pools of twelve BACs, which were sequenced in separate gasket compartments. Single reads of 200 bp were obtained at 30x coverage and were assembled with Newbler software (Roche Applied Science, Almere, The Netherlands). These next generation sequencing BACs were on average 126 kb in length, with on average 13.4 assembled sequence contigs per clone.

Consensus sequence superscaffolds were constructed for the overlapping BAC sequences in a minimal tiling path (MTP) as described [2]. This process employed RH WGS sequence contigs to close kilo base-sized gaps that were present in the BAC assemblies. For 37 MTPs in which the resulting set of sequence scaffolds was still too fragmented for proper evaluation of dot-plot alignments, the scaffolds were placed in the correct order and orientation, based on the BAC order within the tiling path and the physical map, and, where needed and possible, also by alignment to the DM reference genome.

The BAC MTP sequences are available in Additional file 3.

### BAC tiling path construction

BACs for sequencing were selected from the AFLP-fingerprint physical map of genotype RH [20]. With an estimated average clone length of 127 kb and 64478 fingerprinted clones, this diploid physical map has a coverage of 9.6 genome equivalents, which amounts to 4.8 genome equivalents per chromosome haplotype. Theoretically, this physical map should cover 99.2% of each haplotype in the diploid RH genome [64]. In practice, however, many gaps in the physical map posed limits on the lengths of the tiling paths that could be identified.

BAC tiling path sequencing was initiated in clones that were anchored to Chr-5 by haplotype-specific AFLP markers. From these sequenced seed BACs, extension BACs for sequencing were selected from the same fingerprint contig, or from an adjacent contig, based on overlaps in fingerprint pattern or BAC end sequence. During this overlap and extension selection procedure, care was taken that the extension BACs were of the same haplotype as the clones in the tiling path, by verifying that their overlapping BES had a 100% nucleotide match to the tiling path sequence. BACs from 70 initially unanchored BAC contigs were added to the BAC tiling paths, resulting in a total of 211 contigs and 21 singleton BACs of the physical map contributing to the RH Chr-5 sequence.

The selection of tiling path extension BACs was supported by custom software [65] that performed a BLAST alignment of the sequenced BAC clones to the end sequences of the RHPOTKEY library [66]. This web-interfaced BAC-end-tool software was designed to filter out non-specific and low similarity BES hits, and groups the BES that match a clone’s sequence by their fingerprint contig of origin in the BAC physical map. These alignment results were used (1) to verify the identity and contig of the sequenced clone, (2) to find overlapping clones of the same haplotype, and (3) to identify potential fingerprint contigs of the opposite haplotype. This latter identification of allelic BAC contigs proved useful in the collinear euchromatic chromosome regions, where the level of sequence divergence between alternate haplotypes was still moderate, i.e. 97-98% similarity. Non-specific BES matches from unrelated contigs typically reached at most 96% sequence similarity in these regions. In the heterochromatin, the increased sequence diversity between haplotypes nearly always abolished this identification of allelic contigs through BES alignments.

### BAC FISH

Five-colour BAC FISH was performed as described [22]. Briefly, pachytene cell spreads were prepared from anthers of the diploid potato genotype RH. Sheared BAC DNA was labelled with one of five fluorescent dyes and labelled BAC DNAs were pooled in a single hybridization sample, to produce a five-colour BAC hybridisation pattern. The choice of BAC clones and the selection of the alternating colour pattern for the 35-clone BAC ladder FISH was based on preliminary BAC FISH experiments, which had already revealed the approximate order of these BAC clones on Chr-5.

### Sequence alignments

Graphical sequence alignments were performed using MUMmer and Gepard software [67,68] The Gepard alignments were with word lengths of 20 or 25 and at zoom settings of 1:1000 or 1:2000 for general long distance collinearity viewing. Crude estimates of the amount of sequence collinearity between RH BAC tiling paths and DM sequences were measured from the graphical sequence alignment plots with the formula C = 2*O/(L1 + L2), where C is the fraction of collinear sequence, O is the total length of the overlapping sequence segments, and L1 and L2 are the lengths of sequences (or sequence intervals) being compared. In case of full sequence overlap (O = L1 = L2) the collinearity C becomes 1, or 100%.

### Miscellaneous

Gene information for the Chr-5 RH BACs was available at Wageningen University from annotations with the Cyrille2 pipeline software [69]. Gene annotation information of the DM sequences was taken from [70]. Tandem repeats were identified with tandem repeat finder software [71]. GP21 and GP22, which are mentioned in Figure 1, are reference genetic markers in the potato genome [72].

### Availability of supporting data

A list of the names and the GenBank accession numbers of the sequenced RH BAC clones that are included in the RH Chr-5 MTP sequences is given in Additional file 2. The Chr-5 BAC MTP sequences that were used in this publication are provided in Additional file 3.

## Additional files

Additional file 1:
**Addtional statistics of RH BAC tiling paths.** Description of data: Lists specific information of the individual MTPs. Additional file 2:
**RH BAC tiling path compositions.** Description of data: Lists the sequenced BAC clones and their tiling order in the MTPs of potato Chr-5. Additional file 3:
**RH BAC tiling path sequences.** Description of data: Nonredundant, multifasta sequence files of the individual BAC MTPs. The sequence contigs and scaffolds in the MTPs can be either ordered or unordered, depending on the quality status of the tiling path sequence. Additional file 4:
**Regions with structural variation on potato chromosome 5.** Description of data: Lists the regions on Chr-5 where prominent deviations from sequence collinearity were found between aligned RH and DM sequences. Additional file 5: Figure S1. High quality sequence collinearity in potato Chr-5 euchromatin. **Figure S2.** Example of interrupted euchromatic sequence collinearity by non-RGH gene clusters on potato Chr-5. **Figure S3.** Structural variation at the R1 RGH cluster of potato Chr- 5. **Figure S4.** Breakdown of general sequence collinearity in the proximal north euchromatin and north heterochromatin of potato Chr-5. **Figure S5.** Contrasting sequence collinearities in the central heterochromatin of potato Chr-5. Figure S6: Large scale sequence divergence in the central heterochromatin of potato Chr-5.

## Abbreviations

AFLP: Trade mark owned by KeyGene N.V., which covers a PCR-based molecular marker technique that detects SNPs at, or adjacant to, DNA restriction sites
BAC: Bacterial artificial chromosome
BES: BAC-end sequence
BLAST: Basic local alignment search tool
Chr-5: Chromosome 5
CNV: Copy number variation
DAPI: 4′,6-diamidino-2-phenylindole
DM: DM 1–3 516 R44
FISH: Fluorescence in situ hybridisation
FL: Fraction length
Mb: Megabases
kb: Kilobases
MTP: Minimal tiling path
PAV: Presence-absence variation
PGSC: Potato genome sequencing consortium
RGH: Resistance gene homolog
RH: RH89-039-16
SNP: Single nucleotide polymorphism
WGS: Whole genome shotgun
{−}: Haplotype undetermined
{0}: Haplotype 0
{1}: Haplotype 1
