# Supplementary material for: Homologues of potato chromosome 5 show variable collinearity in the euchromatin, but dramatic absence of sequence similarity in the pericentromeric heterochromatin
Source: BMC Genomics. 2015 May 10;16(1):374. doi: 10.1186/s12864-015-1578-1 (PMC4470070; doi:10.1186/s12864-015-1578-1)
Supplement: Additional file 5: Figure S1. — High quality sequence collinearity in potato Chr-5 euchromatin. Figure S2. Example of interrupted euchromatic sequence collinearity by non-RGH gene clusters on potato Chr-5. Figure S3. Structural variation at the R1 RGH cluster of potato Chr- 5. Figure S4. Breakdown of general sequence collinearity in the proximal north euchromatin and north heterochromatin of potato Chr-5. Figure S5. Contrasting sequence collinearities in the central heterochromatin of potato Chr-5. Figure S6: Large scale sequence divergence in the central heterochromatin of potato Chr-5. [file 12864_2015_1578_MOESM5_ESM.pdf]

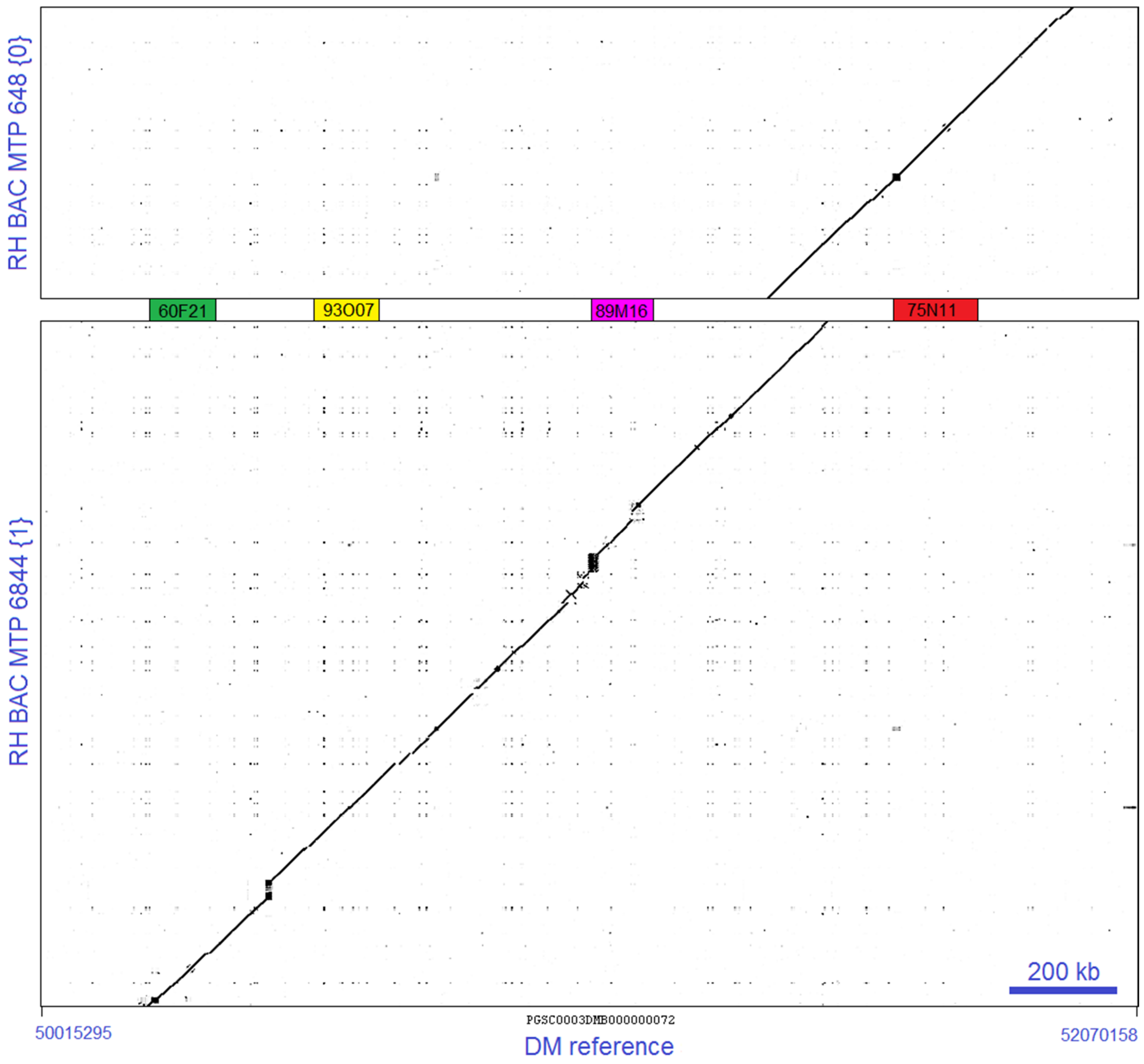

Figure S1. High quality sequence collinearity in potato Chr-5 euchromatin. This alignment of RH MTPs 648 {0} and 6844 {1} to the DM reference sequence shows that a tight, long-distance sequence collinearity exists between both genotypes in the south euchromatin of Chr-5. The RH and DM sequences are approximately 98% similar in this region. The positions of RH BAC clones RH060F21 (green), RH093O07 (yellow), RH089M16 (magenta) and RH075N11 (red), from respectively the homozygous part of MTP 6844 {-}, MTP 429 {0}, the heterozygous part of MTP 6844 {1} and MTP 648 {0}, are indicated in the DM reference sequence. These BAC clones were used for *in situ* hybridisation in Figures 1B and 2.

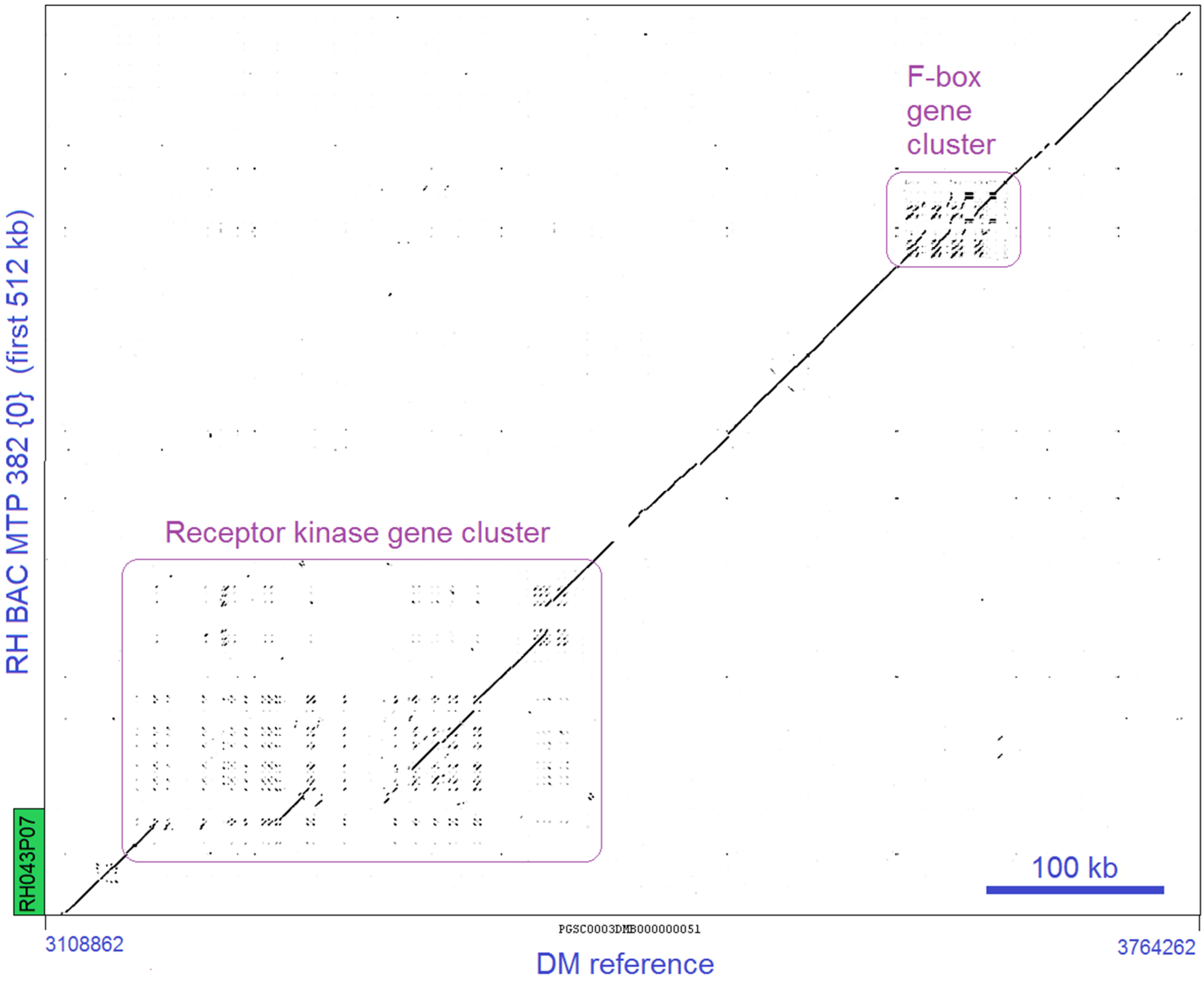

Figure S2. Example of interrupted euchromatic sequence collinearity by non-RGH gene clusters on potato Chr-5. Dot-plot alignment of the first 514 kb of RH BAC MTP 382 {0} to the DM Chr-5 reference sequence in the north euchromatin. The tight sequence collinearity is partially interrupted both at a receptor kinase gene cluster and an F-box gene cluster. The position of BAC clone RH043P07 (green) is shown in MTP 382 {0}. This clone was used for *in situ* hybridization in Figures 1B and 2.

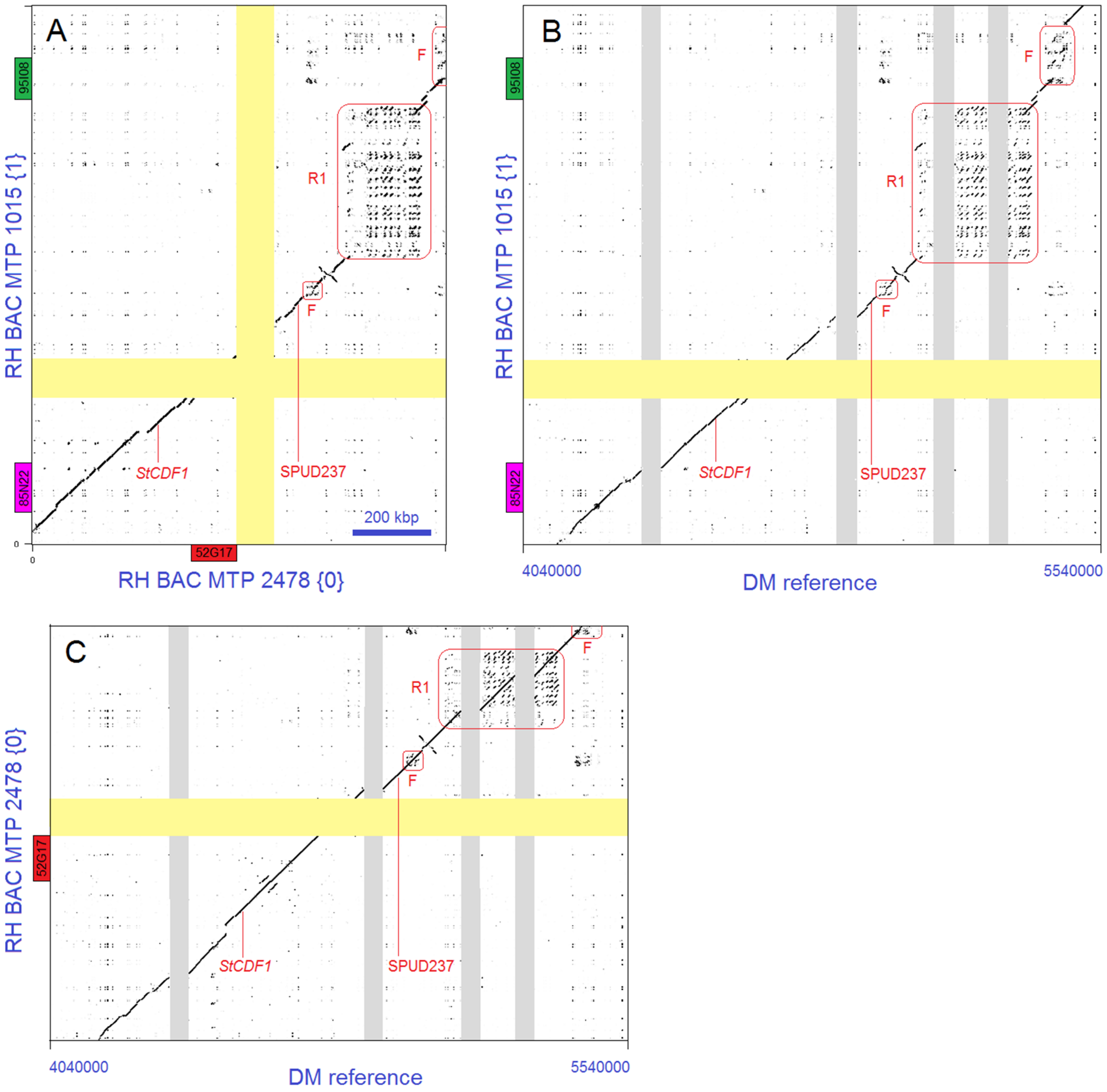

Figure S3. Structural variation at the R1 RGH cluster of potato Chr-5. A. Dot-plot alignment of RH BAC MTP 2478 {0} versus RH BAC MTP 1015 {1}. The two RGH clusters in RH differ in lengh and have no sequence collinearity. **B.** Dot-plot alignment of RH BAC MTP 1015 {1} versus the DM reference genome. Sequence collinearity is interrupted at the R1 cluster and at two F-box gene clusters. **C.** Dot-plot alignment of RH BAC MTP 2478 {0} versus the DM reference genome: haplotype {0} of RH is collinear with DM in R1 cluster and in the smaller F-box gene cluster. **All panels:** The grey bands are gaps in the DM Chr-5 pseudomolecule assembly that were filled with 50 kb of poly-N spacer sequence. Yellow bands indicate unsequenced regions due to gaps in the RH BAC physical map. R1 = late blight RGH cluster; F = F-box gene cluster; *StCDF1* = gene PGSC0003DMG400018408, a key regulator of plant maturity and tuber formation [26]; SPUD237 = commonly used genetic marker for this region. The positions of BAC clones RH085N22 (magenta) and RH095I08 (green) are indicated in MTP 1015 {1}. The position of BAC clone RH052G17 (red) is indicated in MTP 2478 {0}. These three BAC clones were used for *in situ* hybridization in Figures 1B and 2.

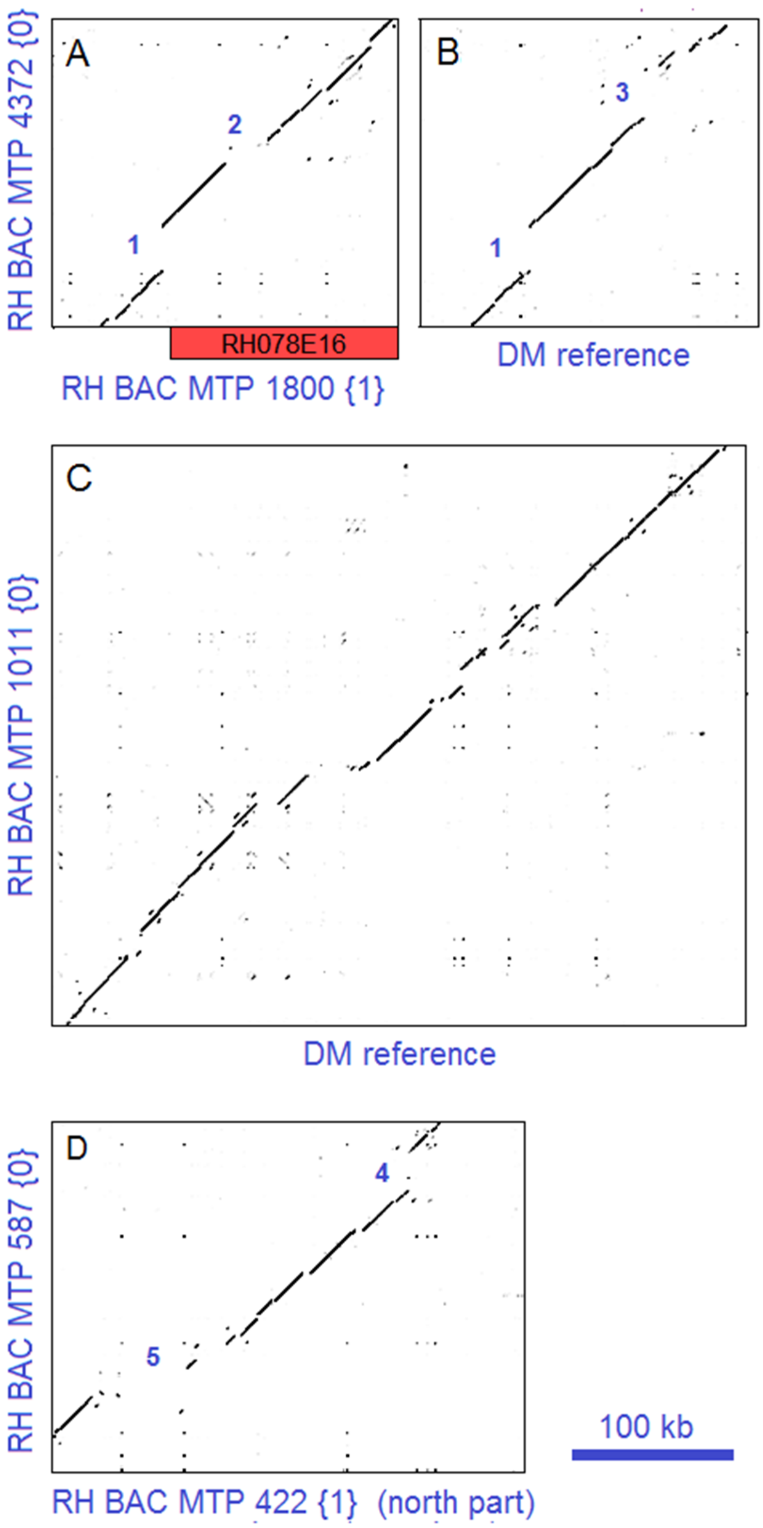

Figure S4. Dot-plot alignments showing breakdown of general sequence collinearity in the proximal north euchromatin and north heterochromatin of potato Chr-5. A, B. Example of insert polymorphisms. RH MTP 4372 {0} has a 28 kb insert (1) in comparison to the homologous regions in RH MTP 1800 {1} and the DM reference genome. MTP 1800 {1} differs from MTP 4372 {0} by a 14 × 27 kb block of non-aligning sequence (2). MTP 4372 {0} differs from DM by a 29 kb insert (3). The position of BAC clone RH078E16 (red) is shown in MTP 4372 {0}. This clone was used for *in situ* hybridization in Figures 1B and 2. **C.** Example of relatively fine interruptions of collinearity in the alignment of RH MTP 1011 {0} to DM. **D.** Polymorphism between RH MTP 587 {0} and the north part of RH MTP 422 {1}, showing an insert of 24 kb (4) and a 30 × 85 kb region of essentially non-aligning sequence between both haplotypes (5). Scale bar of 100 kb applies to all figure panels.

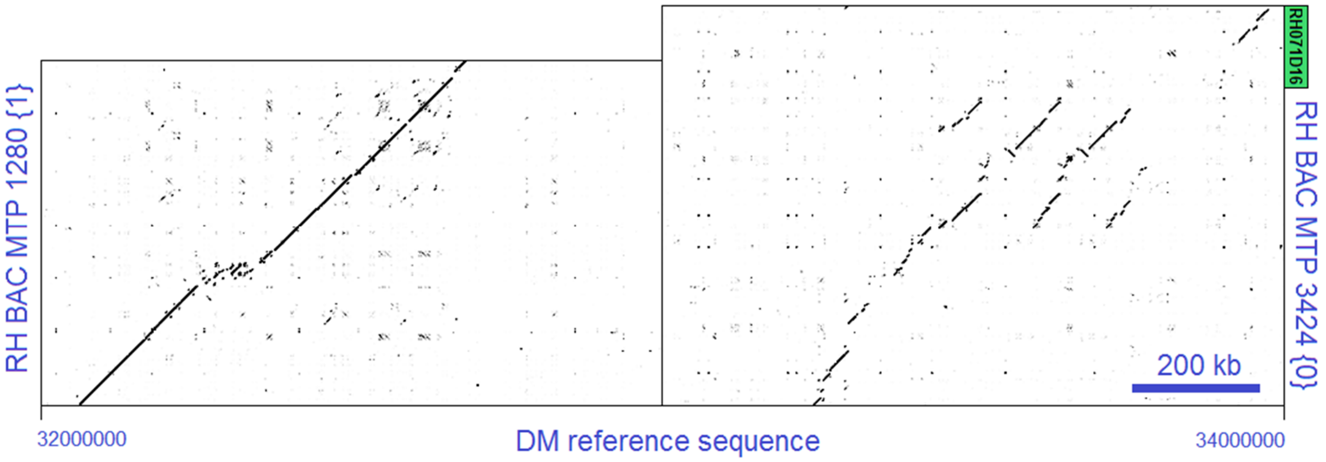

Figure S5. Contrasting sequence collinearities in the central heterochromatin of potato Chr-5. Dot-plot alignment of RH BAC MTPs 1280 {1} and 3424 {0} to DM superscaffold 33 in the central heterochromatin. Within a 2 Mb interval of the DM sequence, a good collinearity exists at MTP 1280 {1}, while the alignment at MTP 3424 {0} is broken, with duplicated regions. The heterochromatin position of clone RH071D16 (green) from MTP 3424 {0} was determined by FISH in Figures 1B and 2.

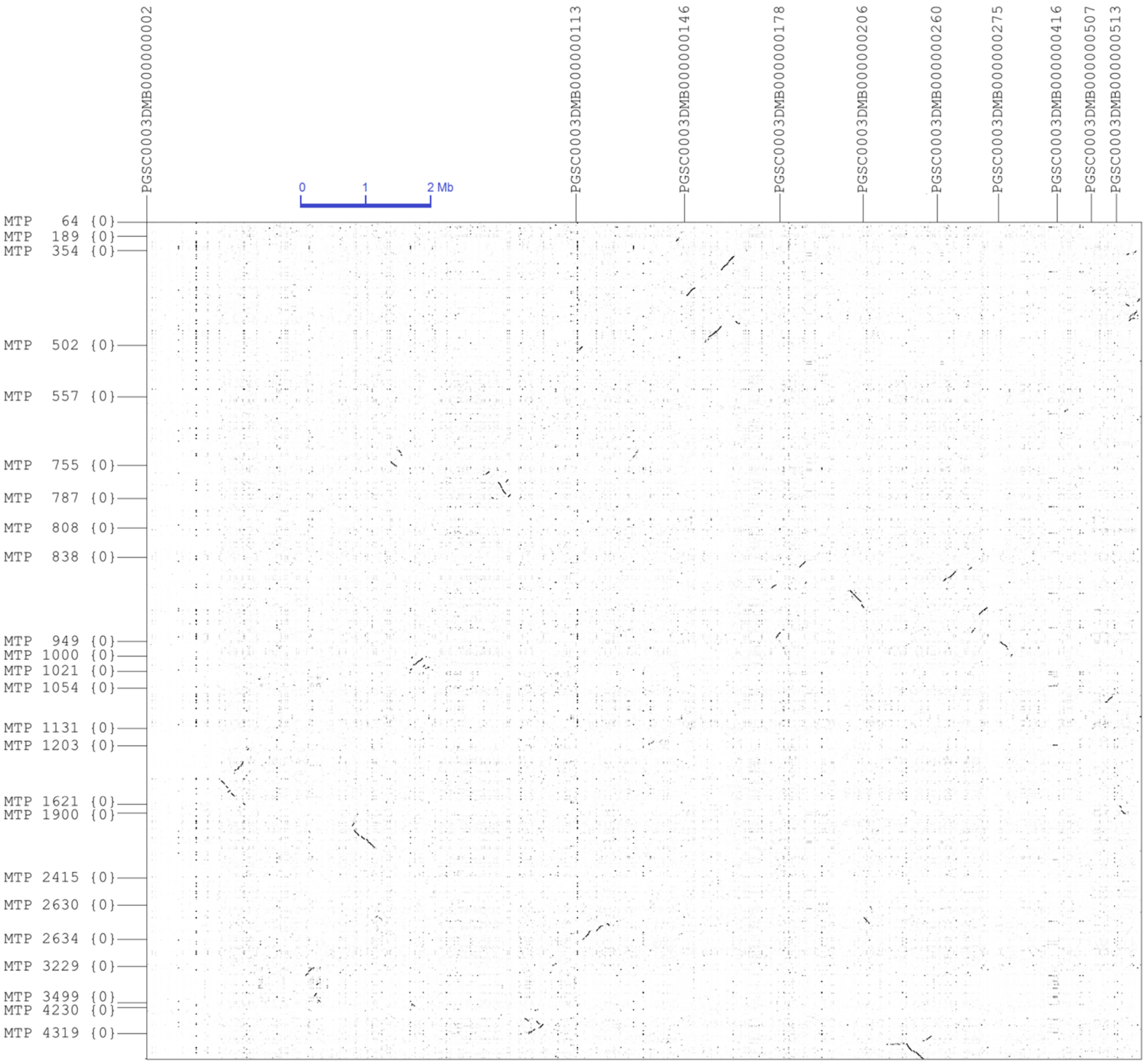

Figure S6. Large scale sequence divergence in the central heterochromatin of potato Chr-5. Dot-plot alignment of concatenated RH haplotype {0} MTP sequences versus concatenated DM sequences from the unaligned sequence region in the central heterochromatin. Only about 36% of the RH haplotype {0} sequences can be retrieved as broken segments of alignment in the DM sequences of this region. In this figure, the DM superscaffold sequences are shown with their full name (i.e. PGSC0003DMB000000XXX). The rightmost three digits correspond with the superscaffold ID numbers in shown in Figure 1C. Similar alignments, with even less overlap, are obtained when comparing the RH haplotype {1} MTPs of this region to DM, or to the RH haplotype {0} MTPs (Table 5).
